# Genome-scale mRNA and small RNA transcriptomic insights into initiation of citrus apomixis

**DOI:** 10.1093/jxb/erw338

**Published:** 2016-09-12

**Authors:** Jian-Mei Long, Zheng Liu, Xiao-Meng Wu, Yan-Ni Fang, Hui-Hui Jia, Zong-Zhou Xie, Xiu-Xin Deng, Wen-Wu Guo

**Affiliations:** Key Laboratory of Horticultural Plant Biology (Ministry of Education), Huazhong Agricultural University, Wuhan 430070, China

**Keywords:** Apomixis, citrus, miRNA, nucellar embryony, ovule, RNA-seq.

## Abstract

Nucellar embryony (NE) is an adventitious form of apomixis common in citrus, wherein asexual embryos initiate directly from nucellar cells surrounding the embryo sac. NE enables the fixation of desirable agronomic traits and the production of clonal offspring of virus-free rootstock, but impedes progress in hybrid breeding. In spite of the great importance of NE in citrus breeding and commercial production, little is understood about the underlying molecular mechanisms. In this study, the stages of nucellar embryo initiation (NEI) were determined for two polyembryonic citrus cultivars via histological observation. To explore the genes and regulatory pathways involved in NEI, we performed mRNA-seq and sRNA-seq analyses of ovules immediately prior to and at stages during NEI in the two pairs of cultivars. A total of 305 differentially expressed genes (DEGs) were identified between the poly- and monoembryonic ovules. Gene ontology (GO) analysis revealed that several processes are significantly enriched based on DEGs. In particular, response to stress, and especially response to oxidative stress, was over-represented in polyembryonic ovules. Nearly 150 miRNAs, comprising ~90 conserved and ~60 novel miRNAs, were identified in the ovules of either cultivar pair. Only two differentially expressed miRNAs (DEMs) were identified, of which the novel miRN23-5p was repressed whereas the targets accumulated in the polyembryonic ovules. This integrated study on the transcriptional and post-transcriptional regulatory profiles between poly- and monoembryonic citrus ovules provides new insights into the mechanism of NE, which should contribute to revealing the regulatory mechanisms of plant apomixis.

## Highlight

Integrative mRNA and small RNA analysis between poly- and monoembryonic ovules reveals involvement of an oxidative stress response and a novel miRNA-target regulatory module in initiation of citrus apomixis.

## Introduction

Apomixis is a type of asexual reproduction, by which clonal embryos are formed in the ovules of flowering plants without female meiosis and fertilization. There are generally two patterns of apomixis depending on whether the embryo is developed from the unreduced female gametophyte (gametophytic apomixis) or directly from the nucellar cells (nucellar embryony, NE) (Spillane *et al.*, 2001; Bicknell and Koltunow, 2004). Apomixis has been reported in over 400 angiosperm species distributed among ~40 plant families (Carman, 1997), but is rarely observed in crop plants (Hand and Koltunow, 2014). NE is common in citrus, one of the most commercially important fruit trees worldwide. The majority of citrus genotypes exhibit genetically inheritable polyembryonic seed development, during which somatic embryos develop from the unreduced nucellar cells along with the zygotic embryo (Aleza *et al.*, 2010; Kishore *et al.*, 2012). NE has long been an obstacle for hybrid breeding of citrus, as the hybrid is morphologically indistinguishable from the clonal offspring derived from one polyembryonic seed. However, offspring developed from nucellar embryos are genetically identical to the female parent, making NE not only important for citrus rootstock propagation but also desirable for trait fixation of field crops. Understanding the mechanisms of NE is expected to provide the theoretical basis for resolving the impediments to citrus hybrid breeding and for introducing NE into crops to maintain desirable traits, such as heterosis.

*Citrus* is a large genus with several major species, among which citron (*Citrus medica*), mandarin/tangerine (*C. reticulata*), and pummelo (*C. grandis*) have been known as the three primitive species (García-Lor *et al.*, 2013), whereas the other species have been recognized as hybrids. Sweet orange (*C. sinensis*) was supposed to be progeny of a backcross between pummelo and mandarin (Xu *et al.*, 2013), whereas grapefruit (*C. paradisi*) has long been recognized as a hybrid between pummelo and sweet orange (de Moraes *et al.*, 2007). Most citrus species are polyembryonic, including grapefruit, most mandarin/tangerine cultivars, and sweet orange; whereas citron, pummelo, and a few mandarin/tangerine cultivars such as clementine are monoembryonic. As one of the most important features of citrus, NE has long provoked great interest among researchers, although the determinant factors that initiate the reprogramming of nucellar cells are still unclear. From previous observations of the developing ovules of Valencia sweet orange, with a large nucleus and deeply stained cytoplasm, the nucellar embryo initial cells (NEI cells) are known to be morphologically distinguishable from the surrounding nucellar cells as early as at anthesis (Koltunow, 1993; Koltunow *et al.*, 1995). In earlier reports, it has been proposed that NE is controlled by either a single, two, or multiple loci (Iwamasa *et al.*, 1967; García *et al.*, 1999; Hong *et al.*, 2001*a*). In later studies, molecular markers related to NE were identified (Nakano *et al.*, 2008*a*, *b*; Kepiro and Roose, 2010). The most recent report on genetics of citrus NE suggested that the candidate genomic region for the polyembryony locus is represented by an approximately 380-kb fragment (Nakano *et al.*, 2012). Using subtractive suppression hybridization (SSH)-based microarrays, two apomictic and three non-apomictic genotype-specific genes have been characterized by comparing the cDNA-catalogue in ovules between the poly- and monoembryonic cultivars at anthesis (Nakano *et al.*, 2013). Applying the same approach, the differentially expressed genes were later profiled in ovules of *Citrus sinensis* at pre- and post-anthesis stages (Kumar *et al.*, 2014). These two studies have provided the first description of transcriptome dynamics during early development of nucellar embryos. With the rapid development of sequencing technology and the release of the genome sequence of two citrus species, namely sweet orange (Xu *et al.*, 2013) and clementine (Wu *et al.*, 2014), RNA-seq has become a promising way to profile the genes involved in NE with higher throughput.

Previous studies have indicated that small RNAs (sRNAs) may play regulatory roles in certain types of apomixis. sRNAs are a class of endogenous single-strand, non-coding RNA with a typical length of 20~24 nucleotides (nt), within which miRNA is the most well-studied category and is known to suppress the expression of target genes at the post-transcriptional level via mRNA cleavage or translational inhibition (Bartel, 2004; Voinnet, 2009). Arabidopsis mutants deficient in proteins required for sRNA biosynthesis and function, such as *ARGONAUTE9* (*ago9*), *SUPPPRESSOR OF GENE SILENCING3* (*sgs3*), *RNA-DEPENDENT RNA POLYMERASE6 (rdr6*), and *ago5*, and the maize mutant *ago104*, have been reported to show phenotypes resembling the morphology of diplospory or apospory (gametophytic apomixis) (Olmedo-Monfil *et al.*, 2010; Singh *et al.*, 2011; Tucker *et al.*, 2012). In the model diplosporous plant *Boechera*, the miR156-targeted transcription factor gene *SQUAMOSA PROMOTER BINDING PROTEIN-LIKE 11* (*SPL11*) was found to be significantly up-regulated in ovules (Amiteye *et al.*, 2011). However, the involvement of sRNA in NE has not yet been determined.

In this study, NEI stage was taken as the key time point when the nucellar cells are reprogrammed and the cell fate is altered. To investigate the genes, sRNA, and molecular processes involved in NEI of citrus, RNA-seq and sRNA-seq were conducted in ovules between two pairs of poly-/monoembryonic ovules prior to and at the appearance of NEI cells. To reduce the genetic background noise, differentially expressed mRNAs and miRNAs were identified between the poly- and monoembryonic ovules within each pair, followed by overlap determination between pairs. After that, we identified the biological processes and pathways enriched in the poly- or monoembryonic ovules. This integrated transcriptome analysis of mRNA and miRNA should enhance our understanding of the NEI process, and it was anticipated that the candidate regulators of NEI would be identified, including the genes and miRNA-target modules.

## Material and methods

### Ovule collection

**Fig. 1.**
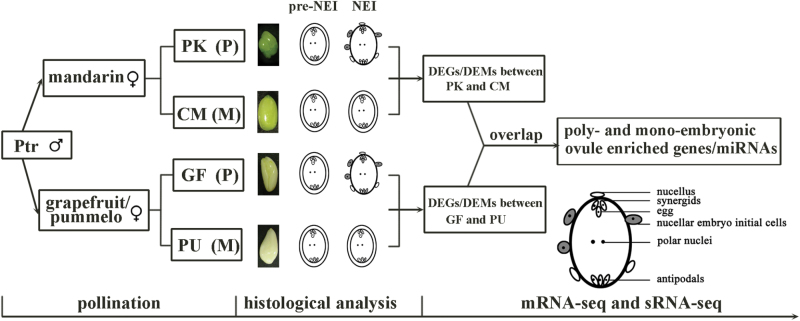
Schematic representation of the experimental design. Ovules collected in 2012 and 2013 were used as two biological replicates. Ptr, precocious trifoliate orange; PK, ‘Huagan No.2’ Ponkan; CM, ‘Nour’ clementine; GF, ‘Cocktail’ grapefruit; PU, ‘Huanong red’ pummelo. P, polyembryonic cultivar; M, monoembryonic cultivar. pre-NEI: the stage immediately before emergence of nucellar embryo initiation (NEI) cells; NEI: the stage at the emergence of NEI cells. DEGs, differentially expressed genes; DEMs, differentially expressed miRNAs. (This figure is available in colour at *JXB* online).

We collected ovules of two pairs of poly-and monoembryonic citrus cultivars. One of the pairs were mandarins comprising the monoembryonic ‘Nour’ clementine (CM, *Citrus clementina*) and the polyembryonic ‘Huagan No.2’ Ponkan (PK, *C. reticulata* Blanco) with as many as 20.6±7.9 embryos per seed. The other pair was pummelo/grapefruit comprising the monoembryonic ‘Huanong red’ pummelo (PU, *C. grandis* (L.) Osbeck) and the polyembryonic ‘Cocktail’ grapefruit (GF, *C. paradisi* Osbeck) with 8.2±4.4 embryos per seed. The adult trees were grown in the germplasm repository at Huazhong Agricultural University (Wuhan, Hubei Province, China). For two consecutive years (2012–2013), the cultivars were hand-pollinated at anthesis with the pollen of early-flowering trifoliate orange (*Poncirus trifoliata*). For each cultivar, ovaries were harvested at anthesis (day 0), and at 3, 7, 14, 21, and 28 days after flowering (DAF ) from three individual trees (Fig. 1). Ovules were dissected from the ovaries under a microscope, followed by immediate immersion in RNAlater (Appiled Biosystems, Foster City, CA, USA). Ovules collected in two consecutive years were taken as biological replicates. mRNA-seq and sRNA-seq sequencing libraries were constructed using samples collected at the stages immediately before and at the emergence of NEI cells.

### Paraffin sectioning

Ovaries and ovules were fixed overnight in FAA (formalin/acetic acid/alcohol), and then dehydrated in 70% ethanol for long-term storage. Fixed tissues were continuously dehydrated using an ethanol series, cleared in xylene and embedded in paraffin wax. The specimens were sectioned to a thickness of 8 μm. Sections were then stained with haematoxylin, and examined and photographed using a BX61 microscope (Olympus, Japan).

### RNA isolation, library preparation, and sequencing

For RNA extraction, each sample contained no less than 1000 ovules. Total RNA of about 10 µg was extracted from each sample using Trizol reagent as described previously (Liu *et al.*, 2006), followed by integrity evaluation using a Bioanalyzer 2100 (Agilent, Germany). The total RNA of each sample was not amplified as there was enough for high-throughput sequencing. mRNA-seq and sRNA-seq libraries were constructed, followed by cluster formation and sequencing using an Illumina Genome Analyzer (CapitalBio Corp, Beijing, China).

### Primary analysis of the sequencing reads

The mRNA and sRNA sequencing raw reads were filtered first by removing the low-quality reads, filtering the contaminants, and trimming the adaptor sequences. We further excluded the sRNA reads with no more than two mismatches to the non-coding RNA in the Rfam database (release 10.1, ftp://ftp.ebi.ac.uk/pub/databases/Rfam), including tRNA, rRNA, snRNA, and snoRNA (Griffiths-Jones *et al.*, 2005). For alignment of all RNA-seq reads of PK and CM, the genome sequence of clementine was used as the reference, because clementine is known to be the hybrid of traditional mandarins and sweet orange (Wu *et al.*, 2014). For GF and PU, the genome sequence of sweet orange was used as the reference, because sweet orange is an interspecific hybrid between pummelo and mandarin (Xu *et al.*, 2013), whereas grapefruit is a hybrid that originated from a cross between pummelo and sweet orange (Li *et al.*, 2010). Tophat (Trapnell *et al.*, 2009) and Bowtie (Langmead, 2010) were used for mapping high-quality reads to the reference genome, with two mismatches (for mRNA reads) and no mismatch (for sRNA reads) allowed. The mapped mRNA transcriptome reads were then assembled with Cufflinks (Trapnell *et al.*, 2010). For the mapped sRNA reads, only those of 20–24 nt with counts ≥2 were retained for further analysis. The raw and processed mRNA and sRNA sequencing data were submitted to the NCBI Gene Expression Omnibus (GEO) under the accession numbers GSE74384 and GSE74132, respectively.

### Identification of miRNAs and targets

The conserved miRNAs were identified by comparing the sRNA reads with the known plant miRNAs registered in miRBase (http://www.mirbase.org/) (Griffiths-Jones *et al.*, 2006) using fasta36 (http://faculty.virginia.edu/wrpearson/fasta/fasta36/), allowing three mismatches. The novel miRNAs were identified from the unmatched reads. The putative miRNA precursors were identified by MIREAP (https://sourceforge.net/projects/mireap/) according to the criteria of Meyers *et al.* (2008). Only those with precursors found in the genome were identified as conserved or novel miRNAs.

The potential miRNA targets were predicted using the online psRNATarget tool (http://plantgrn.noble.org/psRNATarget/) (Dai and Zhao, 2011) with the default parameters. The miRNA sequences were used as the query, whereas the databases were sweet orange transcript sequences (for GF/PU) and clementine transcripts (version 10) retrieved from the Joint Genome Institute (for PK/CM). The two previously published citrus degradome sequencing datasets (GSE46765 and GSE53064) were used to exclude false targets (Liu *et al.*, 2014; Wu *et al.*, 2015). The target genes were annotated using the blast2GO program (Conesa *et al.*, 2005).

### Gene annotation, expression level comparison, and gene ontology enrichment analysis

The unigenes resulted from the assembly of mRNA reads were annotated by comparison with the non-redundant nucleotide and protein database of NCBI using BLASTN and BLASTX, with an *E*-value cut-off of 1*e*^−5^. The expression levels of the mRNA transcripts were measured as fragments per kilobase of exon per million fragments mapped (FPKM), whereas miRNA read counts were normalized to reads per million (RPM). The differentially expressed genes (DEGs) and miRNAs (DEMs) were identified by edgeR (Robinson *et al.*, 2010). The raw read counts of two biological replicates in each sample were submited as input data, then normalized with the default method of trimmed mean of *M*-values (TMM) (Robinson and Oshlack, 2010). The normalization factors were caculated and then the estimate common dispersions of all tags follow by tagwise dispersions. The DEGs and DEMs were determined using the exact test in the package with the false discovery rate (FDR) set as <0.05 and the absolute log_2_-fold change ≥1.0. Benjamini and Hochberg’s algorithm was used to control FDR by default (Benjamini and Hochberg, 1995), and the fold-changes were calculated as: normalized read counts of sample2/sample1. To show the common DEGs expression profile between two pairs of poly-/monoembryonic ovules, heatmaps were generated using pheatmap in the R package (https://cran.r-project.org/web/packages/pheatmap/index.html).

Gene ontology (GO) enrichment analysis was performed using the web-based agriGO program (GO Analysis Toolkit and Database for Agricultural Community) (Du *et al.*, 2010). The DEGs were searched against Arabidopsis protein sequences (ftp://ftp.arabidopsis.org/Proteins/TAIR10_protein_lists/TAIR10_pep_20101214) using BLASTX (*E*-value <1*e*^–3^) to obtain TAIR10 locus IDs, which were submitted to agriGO as a query. Enriched GO terms were analysed using default parameters (FDR<0.05).

### Quantitative RT-PCR procedures

To accurately evaluate the expression levels of the genes in ovules of the four cultivars, the expressional stability of six previously identified housekeeping genes, *ACT7*, *DIM*, *eIF4α*, *UBC21*, *UBL5*, and *UBQ1* (Mafra *et al.*, 2012; Liu *et al.*, 2013), was assessed using Normfinder (Andersen *et al.*, 2004). *U6* was used as the reference gene for miRNAs (Kou *et al.*, 2012). Quantitative real-time PCR (qRT-PCR) was performed and the results were analysed as described previously (Liu *et al.*, 2013). miRNA expression was detected by stem-loop qRT-PCR (Varkonyi-Gasic *et al.*, 2007). Two independent biological replicates and at least three technical replicates were performed. The primer sequences are presented in Supplementary Table S1 at *JXB* online.

### *Nicotiana benthamiana* transient assay

The transient assay in *Nicotiana benthamiana* was performed as previously described (Sparkes *et al.*, 2006) to test whether certain miRNAs repress the predicted target genes *in vivo*. A stem-loop fragment of the miRNA precursor was amplified and transferred into the pK2GW7 vector (Invitrogen) with an empty vector (EV) as the control. The native target sites (5′-CGTCACAA CAGCCTTTTACGG-3′ in *Cs9g06920* and 5′-TGTCATGGGAGTC TGCTACAG-3′ in *orange1.1t00318*), modified target sites (5′-CGTG GGGAAGAAAAGGGGCGG-3′ in *Cs9g06920* and 5′-TGTCATG AAGAGGGGAAGCAG-3′ in *orange1.1t00318*) that could not be cleaved by miRNAs, and perfect complementary sites (5′-GGTCACAGGAGCCTTCTACAG-3′) were inserted into the pMS4 vector (kindly provided by Dr Feng Li, Huazhong Agricultural University, Wuhan, China) carrying green fluorescent protein (GFP). miRNA and its targets were transformed into *Agrobacterium* strain GV3101 and then co-infiltrated into the *Nicotiana benthamiana* leaves. The leaves were harvested 3 d after infiltration and photographed under a hand-held UV light (Beijing, China).

## Results

### Determination of nucellar embryo development stages

**Fig. 2.**
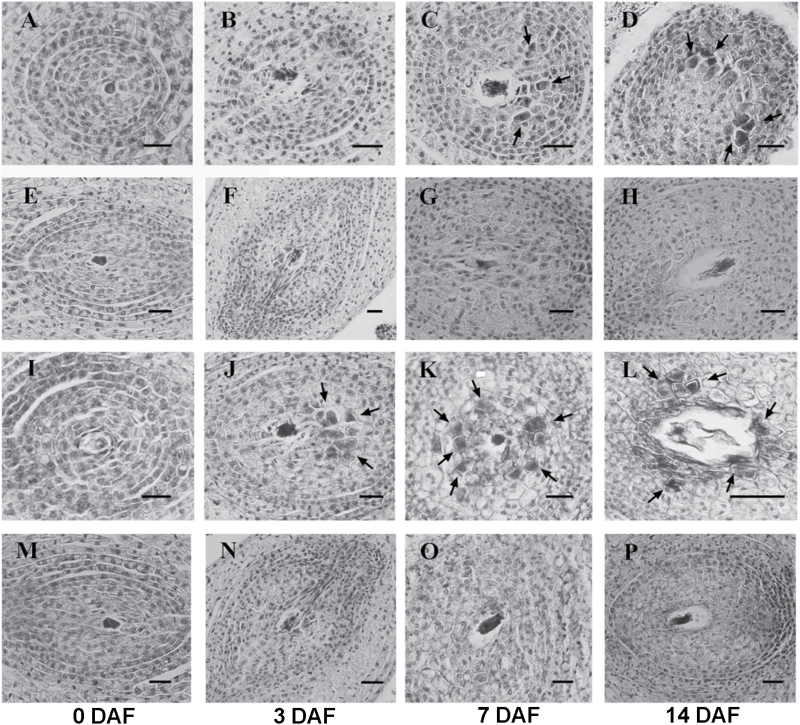
Comparison of nucellar embryogenesis initiation in polyembryonic and monoembryonic ovules at different time points through stained paraffin sections. Ovule samples were harvested from (A–D) ‘Huagan No.2’ Ponkan (PK), polyembryonic cultivar; (E–H) ‘Nour’ clementine (CM), monoembryonic cultivar; (I–L) ‘Cocktail’ grapefruit (GF), polyembryonic cultivar; and (M–P) ‘Huanong red’ pummelo (PU), monoembryonic cultivar, at anthesis (0) and 3, 7, and 14 DAF (days after flowering). The nucellar embryo initiation cells first emerged at 7 DAF in PK (C) and at 3 DAF in GF (J). Arrows indicate NEI cells. Scale bars = 20 μm.

To understand the early events of NE, the time point at which NEI cells are formed was identified by comparing the morphology of the developing nucellar cells between the poly- and monoembryonic cultivars at anthesis (time 0), and at 3, 7, and 14 DAF (Fig. 1). From the stained paraffin section of ovules, the NEI cells surrounding the developing sexual embryo sac could be distinguished from the normal nucellar cells by the presence of condensed cytoplasm, a large nucleus, and thickened cell walls (Fig. 2). No morphological differences were observed in nucellar cells at 0 DAF between poly- and monoembryonic cultivars (Fig. 2); however, the NEI cells emerged in ovules at 3 DAF for ‘Cocktail’ grapefruit and at 7 DAF for ‘Huagan No.2’ Ponkan mandarin (Fig. 2). Therefore, 0 DAF for grapefruit and 3 DAF for Ponkan were taken as the stages just before the emergence of NEI cells, i.e. the ‘pre-NEI’ stage; whereas 3 DAF for grapefruit and 7 DAF for Ponkan were taken as the ‘NEI’ stage. A total of 16 mRNA-seq and 16 sRNA-seq libraries were constructed for the ovules of the two cultivar pairs ‘Huagan No.2’ Ponkan (PK, polyembryonic)/‘Nour’ clementine (CM, monoembryonic) and ‘Cocktail’ grapefruit (GF, polyembryonic)/‘Huanong red’ pummelo (PU, monoembryonic) at the pre-NEI and NEI stages with two biological replicates. For each stage, mRNA and sRNA profiles in ovules were compared between the poly- and monoembryonic cultivars within each pair. The overlapped differentially expressed genes/miRNAs between the two pairs were identified as the factors involved in citrus nucellar embryogenesis (Fig. 1).

### Determination of genes enriched in poly- and monoembryonic ovules

**Fig. 3.**
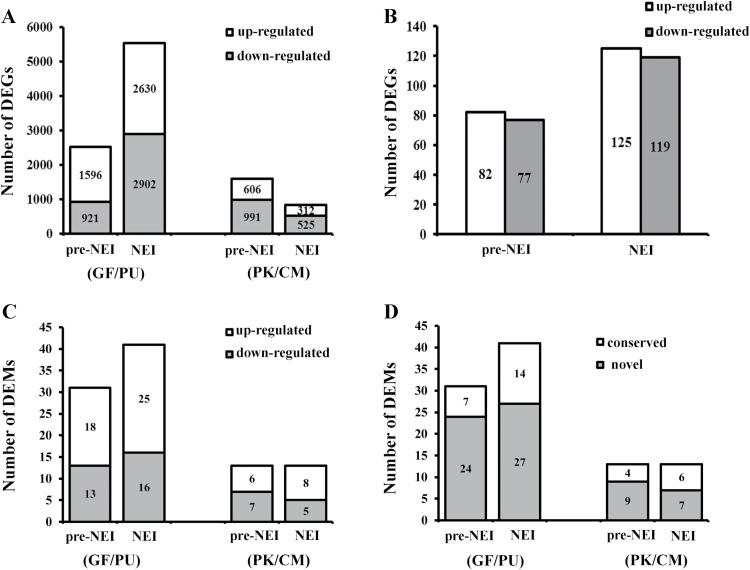
Differentially expressed mRNAs and miRNAs prior to and at nucellar embryogenesis initiation. The numbers of differentially expressed genes (DEGs) and miRNAs (DEMs) at the pre-nucellar embryo initiation (pre-NEI) and NEI stages are shown. The total numbers of DEGs at each time point are labeled within the bars. (A, C) The number of DEGs (A) and DEMs (C) between the poly- and monoembryonic ovules within the cultivar pairs. (B) The number of common DEGs between the poly- and monoembryonic cultivars. (D) The number of differentially expressed conserved and novel miRNAs between the poly- and monoembryonic ovules within the cultivar pairs. PK, ‘Huagan No.2’ Ponkan (polyembryonic); CM, ‘Nour’ clementine (monoembryonic); GF, ‘Cocktail’ grapefruit (polyembryonic); PU, ‘Huanong red’ pummelo (monoembryonic). pre-NEI: the stage immediately before emergence of NEI cells; NEI: the stage at the emergence of NEI cells.

The numbers of reads yielded from the 16 RNA-seq libraries for ovules ranged from 10.8 to 31.5 million. Over 88% of the qualified reads were mapped to the citrus reference genomes (Table 1). Of the 29 455 and 34 011 genes annotated in the sweet orange and clementine gene models, respectively, up to 76.7% (22 595) in GF/PU and 59.9% (20 373) in PK/CM were detected by sequencing during ovule development. The sequencing data of all samples in the two biological replicates were closely clustered, and so were the sequencing data of the same cultivar at different ovule developmental stages, suggesting good correlation between replicates and within the same genotypes (Supplementary Fig. S1). More DEGs were identified in the GF/PU pair than in the PK/CM pair. Specifically, a total of 2517 and 5532 DEGs were identified in the ovules of GF/PU at the pre-NEI and NEI stages, respectively, whereas the corresponding numbers were 1597 and 837 in the ovules of PK/CM (Fig. 3A). Of all the DEGs, 305 were overlapped between the two pairs (Supplementary Table S2), including 159 (82 up-regulated and 77 down-regulated) at pre-NEI and 244 (125 up-regulated and 119 down-regulated) at NEI in the polyembryonic ovules (Fig. 3B). Of these, 98 genes were continuously up- or down-regulated at the two time points. Heatmaps of common DEGs are shown in Supplementary Fig. S2.

**Table 1.** Summary of mRNA-seq data

Sample	Total reads (paired-end)	High-quality reads	Mapped to genome
PK-pre-NEI-br1	12 341 105	23 609 332 (95.65%)	21 949 845 (92.97%)
PK-pre-NEI-br2	10 836 073	20 720 434 (95.61%)	19 169 673 (92.52%)
PK-NEI-br1	11 213 218	21 439 328 (95.60%)	20 004 205 (93.31%)
PK-NEI-br2	11 918 364	22 515 072 (94.46%)	20 205 985 (89.74%)
CM-pre-NEI-br1	11 495 826	21 976 200 (95.58%)	20 809 279 (94.39%)
CM-pre-NEI-br2	15 867 345	28 500 410 (89.81%)	26 830 342 (94.14%)
CM-NEI-br1	11 216 662	21 427 196 (95.52%)	20 288 817 (94.69%)
CM-NEI-br2	11 528 101	21 778 332 (94.46%)	19 666 796 (90.30%)
GF-pre-NEI-br1	11 412 112	21 815 470 (95.58%)	19 794 135 (90.73%)
GF-pre-NEI-br2	31 570 811	52 589 004 (83.29%)	47 579 645 (90.47%)
GF-NEI-br1	11 566 211	22 135 704 (95.69%)	20 121 920 (90.90%)
GF-NEI-br2	17 218 974	30 798 378 (89.43%)	27 728 699 (90.03%)
PU-pre-NEI-br1	11 206 018	21 459 796 (95.75%)	19 324 877 (90.05%）
PU-pre-NEI-br2	13 108 309	24 818 588 (94.67%)	22 004 701 (88.66%)
PU-NEI-br1	11 942 072	22 849 538 (95.67%)	20 342 319 (89.03%)
PU-NEI-br2	14 934 310	26 808 890 (89.76%)	23 767 247 (88.65%)

PK: ‘Huagan No.2’ Ponkan (polyembryonic); CM: ‘Nour’ clementine (monoembryonic); GF: ‘Cocktail’ grapefruit (polyembryonic); PU: ‘Huanong red’ pummelo (monoembryonic). pre-NEI: stage right before emergence of NEI cells; NEI: stage at the emergence of NEI cells. br1/br2: two biological replicates respectively.

**Fig. 4.**
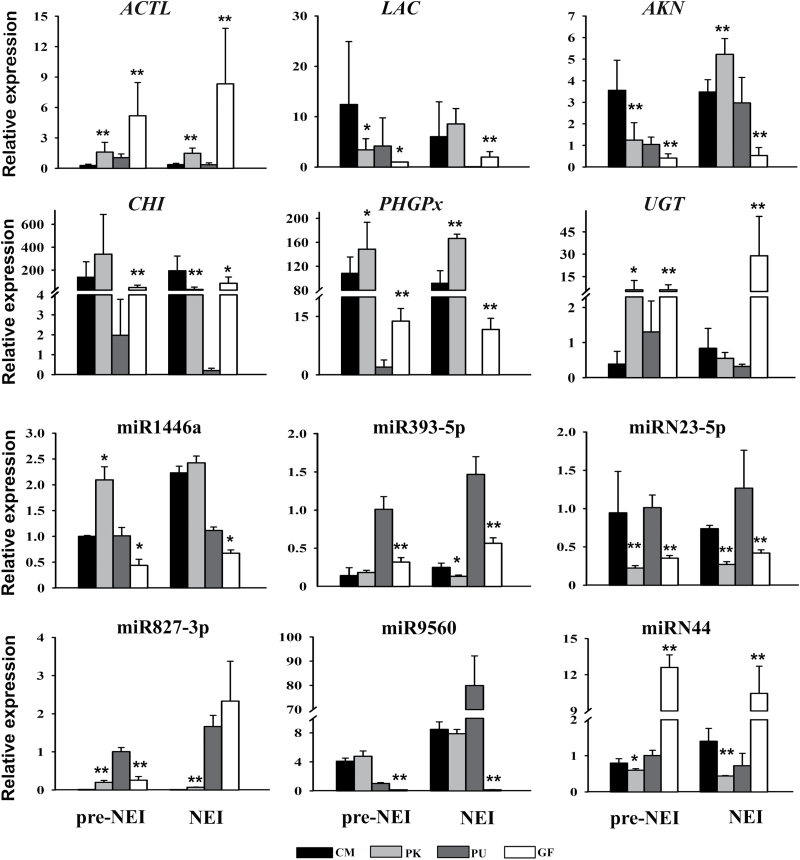
qRT-PCR validation of differential expression patterns of genes and miRNAs in four citrus cultivars prior to and at nucellar embryogenesis initiation. Six mRNAs and six miRNAs were selected for qRT-PCR analysis. *DIM* and *U6* were used as the endogenous controls for mRNA and miRNA, respectively. The error bars indicate standard deviation of three experimental and two years’ biological replicates. PK, ‘Huagan No.2’ Ponkan (polyembryonic); CM, ‘Nour’ clementine (monoembryonic); GF, ‘Cocktail’ grapefruit (polyembryonic); PU, ‘Huanong red’ pummelo (monoembryonic). pre-NEI: stage immediately before emergence of nucellar embryo initiation (NEI) cells; NEI: stage at the emergence of NEI cells. *ACTL*, acidic endochitinase-like; *LAC*, laccase 110; *AKN*, ankyrin repeat family protein; *CHI*, chitinase; *PHGPx*, phospholipid hydroperoxideglutathione peroxidase; *UGT*, UDP-glycosyltransferase. Significant differences in comparison with monoembryonic ovules (the control) are indicated as follows: *P<0.05 and **P<0.01 (Student’s *t*-test).

In light of the results of Normfinder, the *DIM* gene was selected as the reference gene, with stable expression across the ovule samples (Supplementary Fig. S3). The expression levels of six DEGs were detected by qRT-PCR, encoding acidic endochitinase-like (ACTL), laccase 110 (LAC), ankyrin repeat family protein (AKN), chitinase (CHI), phospholipid hydroperoxideglutathione peroxidase (PHGPx), and UDP-glycosyltransferase (UGT) (Fig. 4), and showing expression profiles positively correlated with the deep sequencing results (*R*^2^=0.56).

### Determination of biological processes enriched in poly- and monoembryonic ovules

GO enrichment analysis was performed among DEGs within the separate poly- and monoembryonic ovule pairs before the common GO terms were identified between the pairs, to circumvent differences in the genetic background. As a result, 110 common biological processes were identified to be significantly enriched in the polyembryonic ovules, including 69 up- and 78 down-regulated, with 37 overlapped (Supplementary Fig. S4). At both pre-NEI and NEI stages, the over-represented categories among the up-regulated genes in the polyembryonic ovules were biosynthesis and metabolism processes of phenylpropanoids and flavonoids, as well as responses to oxidative stress, which were enriched in the NEI stage and near-enriched in the pre-NEI stage (FDR=0.06 in the PK/CM pair). Processes enriched among down-regulated genes in the polyembryonic ovules were the salicylic acid (SA) and jasmonic acid (JA) -mediated signaling pathways, as well as responses to stimuli such as temperature, light, sucrose, and disaccharides (Supplementary Fig. S4).

**Fig. 5.**
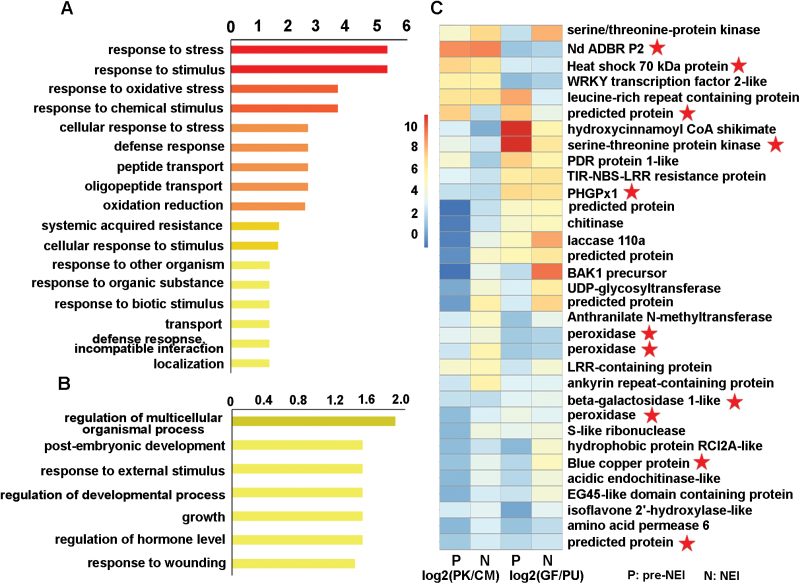
Enriched GO terms in biological processes based on overlapped up-regulated (A) and down-regulated (B) genes, and heatmap (C) of the up-regulated genes in the category ‘response to stress’. GO enrichment analysis was performed based on the 305 overlapped DEGs between the PK/CM and GF/PU pairs at the pre-NEI and NEI stages, with FDR<0.05 as significant. In (A) and (B), the *x*-axis is the –log(FDR value). The red-to-yellow scale represents decreasing significance levels, i.e. red = most, orange = moderate, yellow = least significant. In (C), the red-to-blue scale represents a decreasing log_2_-fold change of gene expression in polyembryonic compared with monoembryonic ovules. Red stars indicate the genes that are also presented in the category ‘response to oxidative stress’. PK, ‘Huagan No.2’ Ponkan (polyembryonic); CM, ‘Nour’ clementine (monoembryonic); GF, ‘Cocktail’ grapefruit (polyembryonic); PU, ‘Huanong red’ pummelo (monoembryonic). P, pre-nucellar embryo initiation (NEI), the stage immediately before the emergence of NEI cells; N, NEI, the stage at the emergence of NEI cells.

To gain a better understanding of the biological processes involved in NEI, GO enrichment analysis was also conducted among the 305 DEGs that overlapped between ovule pairs. The results showed that 17 over-represented biological processes were enriched upon the up-regulated DEGs in the polyembryonic ovules. Among these the majority, and those with the highest confidence, were responses to various stresses and stimuli such as oxidative and chemical stress, in agreement with the up-regulated biological processes shared between ovule pairs. However, only seven terms were significantly enriched among the down-regulated DEGs in the polyembryonic ovules, including post-embryonic development, response to external stimulus, regulation of hormone levels, and response to wounding (Fig. 5). As many as 33 genes were clustered in the polyembryonic ovules enriched category ‘response to stress’, including cell wall modification-related genes (*CHI*, *UGT*, *CTL*, and *LAC*), peroxidase, and heat-shock protein (Fig. 5). Interestingly, ‘response to oxidative stress’ was over-represented using either of the two above-mentioned GO enrichment analyses. Three up-regulated DEGs in polyembryonic ovules encoding peroxidase, as well as *PHGPx* and NADP-dependent alkenal double-bond reductase P2 (*Nd ADBR P2*) genes, were included in the category ‘response to oxidative stress’ (Fig. 5).

### miRNA identification and DEMs between poly-and monoembryonic ovules

For each of the 16 samples, up to 10 million clean reads were generated (Supplementary Table S3). For reads of 18–30 nt, all the libraries showed similar size distributions, with reads of 24 nt being predominant, followed by the reads of 21 nt (Supplementary Fig. S5). An above-average correlation coefficient over 0.8 was found between the two biological replicates (Supplementary Table S4). For miRNA identification, only the reads of 20–24 nt were used (Table 2). A total of 93 conserved mature miRNAs originating from 111 loci were identified in the ovules of the GF/PU pair, belonging to 39 families (Supplementary Table S5). Meanwhile, 91 conserved mature miRNAs originating from 92 loci were identified in the ovules of the PK/CM pair, belonging to 37 families (Supplementary Table S5). The miRNA families identified from the ovules of the four citrus cultivars were almost identical, covering the majority of conserved miRNA families in plants, except for the miR2111 and miR398 families, which were present in the GF/PU pair but absent in the PK/CM pair. In addition, 66 novel miRNAs were identified from 65 genomic loci in the GF/PU pair, whereas 60 novel miRNAs were identified from 58 hairpin precursors in the PK/CM pair (Supplementary Table S5). Among the novel miRNAs, only 20 overlapped between the two pairs of cultivars, accounting for one-third of all the newly annotated miRNAs within each pair.

**Table 2.** Summary of sRNAs of 20–24 nt and counts ≥2 in the 16 libraries

Sample	Clean reads (redundant)	Clean reads (unique)	Matched genome (unique)
PK-pre-NEI-br1	5 264 026	495 628	319 074 (64.38%)
PK-pre-NEI-br2	7 376 087	635 815	448 776 (70.58%)
PK-NEI-br1	6 636 927	718 894	459 566 (63.93%)
PK-NEI-br2	7 379 789	691 272	493 845 (71.44%)
CM-pre-NEI-br1	5 323 341	452 232	321 063 (71.00%)
CM-pre-NEI-br2	3 775 216	313 820	243 737 (77.67%)
CM-NEI-br1	6 414 261	666 383	473 494 (71.05%)
CM-NEI-br2	5 034 185	442 221	348 707 (78.85%)
GF-pre-NEI-br1	7 826 065	739 112	430 509 (58.25%)
GF-pre-NEI-br2	4 699 422	394 642	218 888 (55.46%)
GF-NEI-br1	7 288 803	805 670	462 196 (57.37%)
GF-NEI-br2	6 307 102	661 217	371 075 (56.12%)
PU-pre-NEI-br1	7 919 766	749 177	422 254 (56.36%)
PU-pre-NEI-br2	7 100 630	633 813	332 440 (52.45%)
PU-NEI-br1	9 917 758	1 037 534	587 302 (56.61%)
PU-NEI-br2	9 361 909	864 776	451 708 (52.23%)

PK, ‘Huagan No.2’ Ponkan (polyembryonic); CM, ‘Nour’ clementine (monoembryonic); GF, ‘Cocktail’ grapefruit (polyembryonic); PU, ‘Huanong red’ pummelo (monoembryonic). pre-NEI, stage immediately before emergence of NEI cells; NEI, stage at the emergence of NEI cells. br1 and br2, biological replicates 1 and 2.

Compared with the monoembryonic cultivar PU, 18 miRNAs were up-regulated and 13 miRNAs were down-regulated in ovules of the polyembryonic cultivar GF at the pre-NEI stage. Meanwhile, 41 miRNAs were differentially expressed at the NEI stage, with 25 up- and 16 down-regulated in the GF ovules (Fig. 3C, Supplementary Table S6). However, only 13 miRNAs showed differential expression at the pre-NEI stage within the PK/CM pair, with six up- and seven down-regulated in ovules of the polyembryonic cultivar PK. At the NEI stage, eight miRNAs were up-regulated whereas five were down-regulated in PK (Fig. 3C, Supplementary Table S6). Interestingly, among the differentially accumulated miRNAs between poly- and monoembryonic ovules, the majority were novel rather than conserved miRNAs (Fig. 3D). Almost every differentially expressed miRNA identified at the pre-NEI stage between poly- and monoembryonic ovules also showed differential expression at the NEI stage. However, no common DEMs with consistent expression patterns were found between PK/CM and GF/PU under the expected criteria with a fold-change of 2.0 and FDR of 0.05. Only the conserved miR1446a and the novel miRN23-5p were consistently down-regulated in the polyembryonic ovules at both time points when FDR was set as 0.06. As determined by stem-loop qPCR, miRN23-5p was down-regulated in the polyembryonic ovules, which was consistent with high-throughput sequencing data. However, miR1446a was down-regulated in GF/PU, but up-regulated in PK/CM (Fig. 4). In addition, another four DEMs were selected to be validated by qPCR. As shown in Fig. 4, miR393b-5p was down-regulated in the polyembryonic ovules in GF/PU at both stages, but only at the NEI stage in PK/CM. miR827-3p was up-regulated in PK/CM, which was consistent with sRNA-seq data. miR9560 showed extremely high expression in PU at the NEI stage, and very low detection in GF at both stages. Similar to the high-throughput sequencing data, miRN44 displayed contrary expression patterns in the two pairs, being down-regulated in PK/CM and up-regulated in GF/PU. Overall, the expression profiles of these six miRNAs exhibited positive correlations between deep sequencing and qRT-PCR (*R*^2^=0.64).

### miRNA–mRNA interaction identified in NEI

A total of 417 and 152 targets were identified for the conserved miRNAs and novel miRNAs, respectively, in the GF/PU pair; however, for the PK/CM pair the corresponding numbers were 695 and 163 (Supplementary Table S7). Although the number of identified targets was different, the target annotation of the conserved miRNA families was almost the same between the two pairs. Fewer targets were identified for the novel miRNAs than for the conserved miRNAs. Most interestingly, some novel miRNAs targeted the same mRNA families as the conserved miRNAs. For example, miRN04 and miR394 targeted *F-box* genes in the GF/PU pair, whereas miR166 and miRN48 targeted *HD-Zip* genes in the PK/CM pair.

To identify the potential miRNA–mRNA regulatory network involved in NEI, the expression patterns of 77 genes in the GF/PU pair and 55 genes in the PK/CM pair targeted by the DEMs were retrieved from the RNA-seq data (Supplementary Table S8). As miRNAs are known to mediate the cleavage of target mRNA, miRNAs and their corresponding targets are supposed to have reciprocal expression patterns. In this study, however, not only negative but also positive correlations in expression levels were observed between miRNAs and their targets. For the GF/PU pair, only three negatively correlated and eight positively correlated miRNA-target modules were found at the pre-NEI stage, whereas 15 negatively correlated and 20 positively correlated miRNA-target modules were found at the NEI stage. For the PK/CM pair, five negatively correlated and five positively correlated miRNA-target modules were found at the pre-NEI stage, whereas eight negatively correlated and four positively correlated miRNA-target modules were found at the NEI stage.

**Fig. 6.**
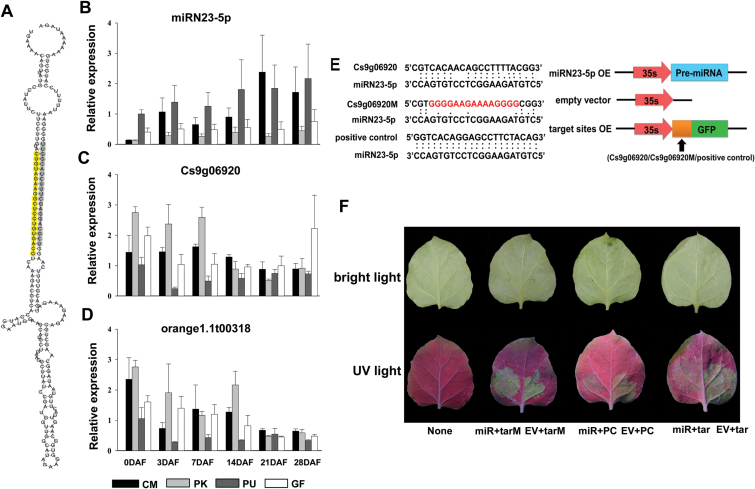
Validation of miRN23-5p-directed cleavage of Cs9g06920 and the expression patterns of miRN23-5p-targets detected by qRT-PCR. (A) Secondary structure of miRN23-5p. The mature sequence located at the 5′ end of the precursor is highlighted in yellow and the complementary miRNA* at the 3′ end is shaded grey. (B) Expression of miRN23-5p at 0, 3, 7, 14, 21, and 28 DAF (days after flowering) in the four cultivars, with *U6* as the endogenous control. (C, D) Expression of target transcripts at 0, 3, 7, 14, 21, and 28 DAF in the ovules of the four cultivars. The error bars indicate the standard deviation of three experimental and two years’ biological replicates. PK, ‘Huagan No.2’ Ponkan (polyembryonic); CM, ‘Nour’ clementine (monoembryonic); GF, ‘Cocktail’ grapefruit (polyembryonic); PU, ‘Huanong red’ pummelo (monoembryonic). (E) Overexpression vectors constructed for transient expression in *Nicotiana benthamiana*. miRN23-5p-OE, miRN23-5p overexpression construct; empty vector, the negative control; target sites OE, construct overexpressing the native target sites (Cs9g06920), the modified target sites insensitive to miRN23-5p (Cs9g06920M); positive control, construct overexpressing target sites perfectly matched to miRN23-5p. (F) Co-infiltrated leaves and the control photographed 3 d after infiltration under either bright light or UV light. All the leaves except the first one without any treatment were co-infiltrated with miRN23-5p (left side of leaf) or empty control (right side of leaf) along with either native target Cs9g06920, modified target Cs9g06920M, or positive control. None, no co-infiltration, negative control; miR, miRN23-5p; tar, native target Cs9g06920; tarM, modified target Cs9g06920M; PC, positive control.

Interestingly, miRN23-5p and its targets were reciprocally expressed during the early stages of nucellar embryo development. miRN23-5p was a novel miRNA derived from the 5′ end of its precursor, which forms a nearly perfect stem-loop structure with no mismatch or bulge between the mature miRNA and the complementary miRNA* (Fig. 6A). qRT-PCR analysis results showed that over two successive years, miRN23-5p was consistently accumulated at a higher level in the ovules of the monoembryonic cultivars than in the polyembryonic cultivars at six time points immediately before, at, and post-NEI (Fig. 6B). In addition, the difference in the expression level of miRN23-5p between the mono- and poly-embryonic ovules became more pronounced at the later stages. A negative correlation in expression was found between miRN23-5p and its target genes in the early stages. Among the three targets of miRN23-5p, Cs9g06920 (Ciclev10005503m.g), which is annotated as an unknown protein, showed a reverse expression pattern to miRN23-5p from anthesis to 7 DAF, with a higher accumulation in the polyembryonic ovules than in the monoembryonic counterparts (Fig. 6C). Another target, orange1.1t00318 (Ciclev10023521m.g), which is annotated as digalactosyldiacylglycerol (DGDG) synthase, shared a similar expression pattern with Cs9g06920 from anthesis to 14 DAF (Fig. 6D). To verify the interaction between miRN23-5p and the two potential targets *in vivo*, a transient assay was conducted in tobacco leaves. The results showed that miRN23-5p mediated cleavage of the target transcript Cs9g06920, but not orange1.1t00318. This was determined based on the significant reduction of GFP fluorescent signal intensity that resulted from co-expression of miRN23-5p with perfectly complementary of the native target site of Cs9g06920 but not of orange1.1t00318, compared with co-expression with the modified insensitive target sites or the empty vector tranformants (Fig. 6E, F and Supplementary Fig. S6).

## Discussion

In this study, we performed an integrated mRNA and miRNA high-throughput sequencing analysis in the polyembryonic (apomictic) and monoembryonic (sexual) ovules in citrus, as a follow-up to the findings of two previous studies (Nakano *et al.*, 2013; Kumar *et al.*, 2014), in order to further our understanding of citrus apomixis, which presents a fascinating developmental problem. As nearly isogenic lines (NILs) for this trait are hardly found in citrus, two pairs of poly- and monoembryonic ovules were used for sequencing, which it was anticipated would successfully reduce the background noise. From the respective and common DEGs between ovule pairs we characterized several biological processes that differed between poly- and monoembryonic ovules, and in addition also identified the miRNA-mediated regulatory pathway related to NEI.

We successfully identified DEGs in two pairs of poly-/monoembryonic ovules, and the common DEGs between these two pairs were also determined. As the genetic difference in the GF/PU pair was larger than that of the PK/CM pair, many more DEGs were identified in GF/PU than in PK/CM at both the pre-NEI and NEI stages (Fig. 3). Recently, the genomic DNA region potentially governing citrus polyembryony has been reported (Nakano *et al.*, 2012), and is a 380-kb fragment containing 70 ORFs. In this study, only one of these ORFs, Cs4g05910, which encodes an ankyrin repeat family protein, was consistently down-regulated in polyembryonic ovules at pre-NEI stage (Fig. 4). Ankyrin repeat family proteins have important and diverse biological roles related to anti-oxidation metabolism in disease resistance and stress responses (Yan *et al.*, 2002), and cell differentiation and development (Zhang *et al.*, 1992). The Arabidopsis *EMB506* gene, which encodes an ankyrin repeat-containing protein, has been shown to be essential for normal zygotic embryo development (Albert *et al.*, 1999), but the regulatory mechanism remains unknown so far. The decreased expression of ankyrin repeat family proteins in the polyembryonic ovules indicated its distinct role in nucellar embryogenesis compared to zygotic embryogenesis.

### Activation of a stress-response process might be required for NEI

It has been proposed that somatic embryogenesis (SE) is an extreme stress response of *in vitro* cultured plant cells (Pasternak *et al.*, 2002), which respond to exogenous or endogenous signals that force a developmental switch (Fehér, 2015). In this study, the GO term ‘response to stress’ was enriched in polyembryonic ovules, irrespective of whether this was based on the overlapped enriched terms between two pairs or on identification of GO terms upon common upregulated genes, and this was in agreement with a previous report on citrus polyembryony (Kumar *et al.*, 2014), suggesting that a stress response would probably be required for induction of nucellar embryogenesis, a form of SE *in vivo*. As the ovule samples were collected at the same time and under the same conditions, we therefore suggest that the stress response was triggered by an endogenous rather than exogenous stimulus.

Stress factors, including osmosis, heavy metal ions, and dehydration, have been well characterized to induce SE in Arabidopsis (Ikeda-Iwai *et al.*, 2003), carrot (Kiyosue *et al.*, 1990), and common bean (Cabrera-Ponce *et al.*, 2015). As an oxidative stress response was identified to be exclusively enriched in polyembryonic ovules, we infer that NEI cells might undergo oxidative stress to gain the competence to induce nucellar embryogenesis. Induced oxidative stress has been shown to promote SE, whereas excess oxygen uptake in turn increases the activity of antioxidant enzymes, such as superoxide and peroxidase (Ganesan and Jayabalan, 2004). In our study, the genes encoding peroxidase and PHGPx were significantly up-regulated in polyembryonic ovules (Fig. 5, Supplementary Table S2), and the GO term ‘oxidative stress responses’ was enriched among up-regulated DEGs in polyembryonic ovules, indicating that oxidative stress responses might promote nucellar embryo initiation and induced expression of antioxidant enzymes. Key issues that should be addressed in the future concern how the endogenous oxidative stress is induced in polyembryonic ovules and how NEI is promoted by the oxidative stress response.

Stress responses would yield changes at multiple levels, including membrane composition, cell wall modifications, as well as secondary metabolite adjustment (Fehér, 2015). Among the 33 genes represented in the ‘response to stress’ category in our study, four genes involved in cell wall modification, *UGT*, *LAC*, *ACHI*, and *CHI*, were found to be up-regulated in the polyembryonic ovules compared to the monoembryonic ovules (Figs 4 and 5). In a previous study, genes involved in plant cell wall modification were also found to be enriched in the apomictic initial cells of *Boechera gunnisoniana* compared to the surrounding sporophytic nucellus tissue (Schmidt *et al.*, 2014). *UGT* may act as part of a callose synthase complex that transfers UDP-glucose to form callose (Hong *et al.*, 2001*b*), which has been reported to accumulate in the zygotic and somatic embryonic cells at the early embryogenesis stage (Leljak-Levanić *et al.*, 2015). Callose deposition in the cell wall has long been proposed as a way to create a physical and molecular barrier that permits somatic cells to reprogram (You *et al.*, 2006; Tao *et al.*, 2012; Parra-Vega *et al.*, 2015). In the present study, the differentiated NEI cells were also found to be isolated from the surrounding nucellar cells by a thickened cell wall, which might have resulted from the highly expressed *UGT* gene in the polyembryogenic ovules at the NEI stage (Fig. 5). Laccase is normally necessary for lignin polymerization in specific cell types with thickened cell walls (Zhao *et al.*, 2013). In our study, the identified *LAC* genes showed consistently higher expression in the polyembryogenic ovules at the NEI stage than in the mono-embryogenic counterparts, which indicates that cell wall modification mediated by laccase might also contribute to NEI.

### Possible roles of miRNAs in NEI

In citrus, somatic embryo induction from callus *in vitro* and nucellar embryogenesis *in vivo* are considered to be two forms of SE. Increasing attention has been paid to the role of miRNAs in SE in plants, including cotton (Yang *et al.*, 2013), citrus (Wu *et al.*, 2015), and Arabidopsis (Wójcik and Gaj, 2016). In our study, about 150 miRNAs (including conserved and novel miRNAs) were identified in each pair of poly- and monoembryonic ovules (Supplementary Table S5). Few significantly differentially expressed miRNAs were found to overlap between GF/PU and PK/CM, which might be due to the limited involvement of miRNAs in NEI. In addition, as a consequence of using ovule samples to prepare sequencing libraries the differential expression profile of genes between NEI cells and somatic nucellar cells was inevitably underestimated.

miR156 has been reported to accumulate abundantly in embryogenic callus and during somatic embryogenesis of citrus (Wu *et al.*, 2015). In the diplosporous species *Boechera*, the transcription factor gene *SQUAMOSA PROMOTER BINDING PROTEIN-LIKE 11* (*SPL11*), which is targeted by the miR156/157 family, was found to be up-regulated at the megaspore mother cell stage of ovule development (Amiteye *et al.*, 2011). However, the miR156/157 family was found to express at low levels in citrus ovules, and the majority of the *SPL* genes showed non-differential expression between poly- and monoembryonic ovules, implying that the miR156/157-*SPL* module might not be involved in nucellar embryogenesis in the way that it is in citrus SE and diplosporous apomixis.

miRN23-5p, a 21-nt novel miRNA derived from a precursor with a nearly perfect secondary structure and with a relatively high read count, was down-regulated in the polyembryonic ovules during development, whereas the corresponding target, Cs9g06920, showed a reciprocal expression pattern at early developmental stages (0–7 DAF) (Fig. 6). Cs9g06920 (Ciclev10005503m.g) is annotated as an unknown protein with a conserved XS domain. An Arabidopsis mutant for one of the XS domain-containing proteins, *SUPRESSOR OF GENE SILENCING 3* (*SGS3*), showed a phenotype resembling aposporic apomixes (Olmedo-Monfil *et al.*, 2010). Since the function of the Cs9g06920-encoded protein is unknown, the role of the ‘miRN23-5p-Cs9g06920’ module in NEI remains unclear at present. However, since *SGS3* encodes a putative RNA-binding protein that functions in the biosynthesis of trans-acting small interfereing RNA (tasiRNA) (Peragine *et al.*, 2004), we suggest that sRNA-mediated epigenetic regulation might also be involved in NEI.

### Comparison of nucellar embryogenesis with somatic embryogenesis from callus

As concluded from previous reports and this present study, nucellar embryogenesis occurring *in vivo* shares quite a few commonalities and yet also shows drastic differences with citrus SE from *in vitro* cultured callus. NEI cells and embryogenic callus cells are both characterized by a large nucleus, dense cytoplasm, and thickened cell walls (Koltunow *et al.*, 1995; Ge *et al.*, 2012), which are morphological markers indicating embryogenic competence. Enhanced callose deposition is responsible for the thickened cell wall, which isolates embryogenic cells from the surrounding somatic cells to initiate cell fate reprograming. Correspondingly, the callose synthase gene was found to be up-regulated in the apomictic ovules in this present study.

Reprogramming of both types of somatic cells requires either environmental stimuli or endogenous signals. SE *in vitro* is well known to be triggered by stress (Rose *et al.*, 2013; Jin *et al.*, 2014), especially by oxidative stress derived from reactive oxygen species (ROS) (Ganesan and Jayabalan, 2004; Cheng *et al.*, 2015). In our study, stress response processes, especially the oxidative stress response, were also suggested to be induced for NEI. Stress response genes and proteins have also been identified previously to be up-regulated during citrus SE (Pan *et al.*, 2009; Ge *et al.*, 2012), including PHGPx and peroxidase as found in the polyembryonic ovules in this present study, as well as copper/zinc superoxide dismutases. We propose that the early oxidative stress prior to nucellar embryonic cell fate determination and the later anti-oxidative stress response of oxygen scavenging may act to fine-tune intracellular redox homoeostasis during nucellar embryo intiation.

*LEC1/B3* regulatory network genes have been reported to play important roles in citrus SE in *vitro* (Ge *et al.*, 2012); however, they were not differentially expressed between poly- and monoembryonic ovules. The conserved miR156a/b, miR164b, and miR171c have been found to be abundantly accumulated in embryogenic tissues and during the SE process (Wu *et al.*, 2015); however, none of these miRNAs was found to be differentially expressed between the poly- and monoembryonic ovules (Supplementary Table S6).

In conclusion, our study provides the first integrated mRNA-seq and sRNA-seq analysis of citrus ovules to explore the candidate genes and miRNAs that may regulate citrus apomixis. NEI was found to be associated with several biological processes, especially the activation of oxidative stress responses. The novel miRN23-5p-Cs9g06920 module was identified as acting to fine-tune the regulation at the post-transcriptional level. This study thus provides new insights into the regulatory mechanisms of nucellar embryony of citrus, and should be of value to future studies of plant apomixis.

## Supplementary data

Supplementary data are available at *JXB* online.

Figure S1. Heatmap clustering of mRNAs.

Figure S2. Heatmap of log_2_-transformed fold-change of differentially expressed genes in the PK/CM and GF/PU pairs.

Figure S3. Selection of suitable reference genes for mRNA expression normalization.

Figure S4. The overlapped enriched GO terms (biological processes) in the PK/CM and GF/PU pairs.

Figure S5. Length distribution of sRNA in the 16 sequence libraries.

Figure S6. A transient assay in *Nicotiana benthamiana* could not validate miRN23-5p directed cleavage of the potential target orange1.1t00318.

Table S1. Primers used for qRT-PCR and the transient assay.

Table S2. Identification of common differentially expressed genes between poly- and monoembryonic ovules.

Table S3. Summary of data statistics of sRNA libraries.

Table S4. Correlation coefficients between biological replicates of sRNA-seq.

Table S5. Identification and expression of known and novel miRNAs in the poly- and monoembryonic ovules.

Table S6. Differentially expressed miRNAs identified between the poly- and monoembryonic ovules within cultivar pairs.

Table S7. miRNA targets identified in ovules of the two pairs of poly- and monoembryonic cultivars.

Table S8. Differentially expressed miRNAs and their corresponding targets extracted from RNA-seq.

Supplementary Data
