# Supplementary material for: Genome-scale mRNA and small RNA transcriptomic insights into initiation of citrus apomixis
Source: J Exp Bot. 2016 Sep 12;67(19):5743–56. doi: 10.1093/jxb/erw338 (PMC5066493; doi:10.1093/jxb/erw338)
Supplement: Supplementary Data [file supp_67_19_5743__index.html]

Genome-scale mRNA and small RNA transcriptomic insights into initiation of citrus apomixis — Genome-scale mRNA and small RNA transcriptomic insights into initiation of citrus apomixis — Supplementary Data 

# Genome-scale mRNA and small RNA transcriptomic insights into initiation of citrus apomixis

## Supplementary Data

Data files

- Supplementary\_Figures\_S1\_S6\_Tables\_S1\_S3\_S6\_S8.pdf - Supplementary Data
- Supplementary\_Table\_S2\_S7.xlsx - Supplementary Data
