# Supplementary material for: Genome-scale mRNA and small RNA transcriptomic insights into initiation of citrus apomixis
Source: J Exp Bot. 2016 Sep 12;67(19):5743–56. doi: 10.1093/jxb/erw338 (PMC5066493; doi:10.1093/jxb/erw338)
Supplement: Supplementary Data [file supp_erw338_Supplementary_Figures_S1_S6_Tables_S1_S3_S6_S8.pdf]

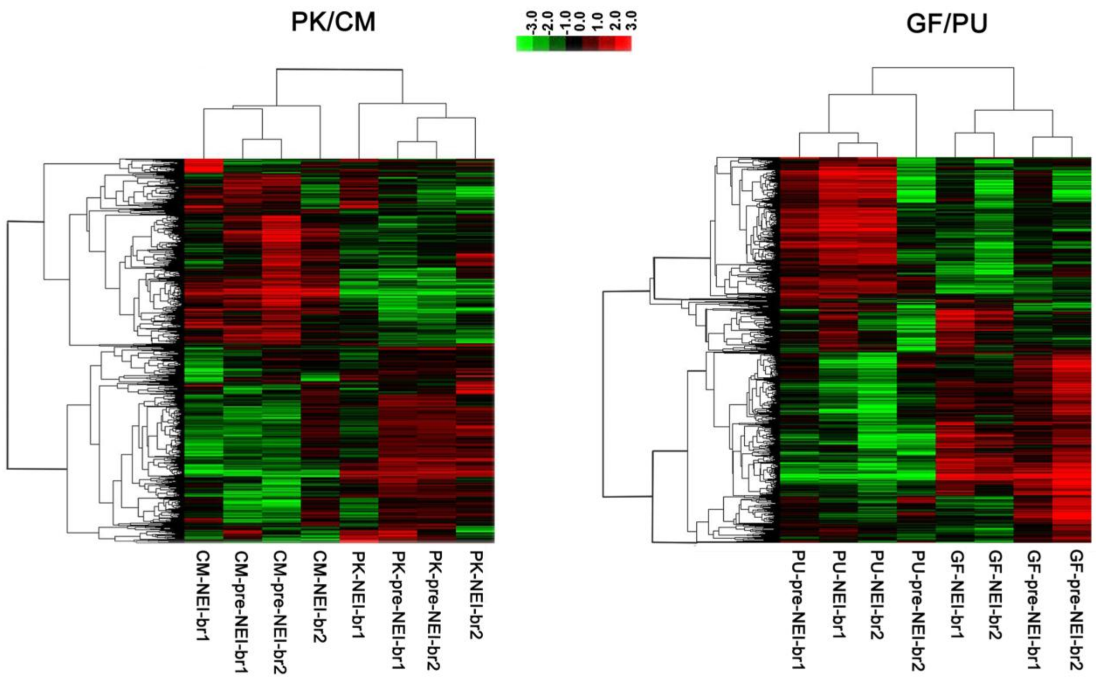

Figure S1 Heatmap clustering of mRNAs. PK: 'Huagan No.2' Ponkan (polyembryonic); CM: 'Nour' clementine (monoembryonic); GF: 'Cocktail' grapefruit (polyembryonic); PU: 'Huanong red' pummelo (monoembryonic). pre-NEI: stage right before the emergence of NEI cells; NEI: stage at the emergence of NEI cells. br1/br2: two biological replicates respectively.

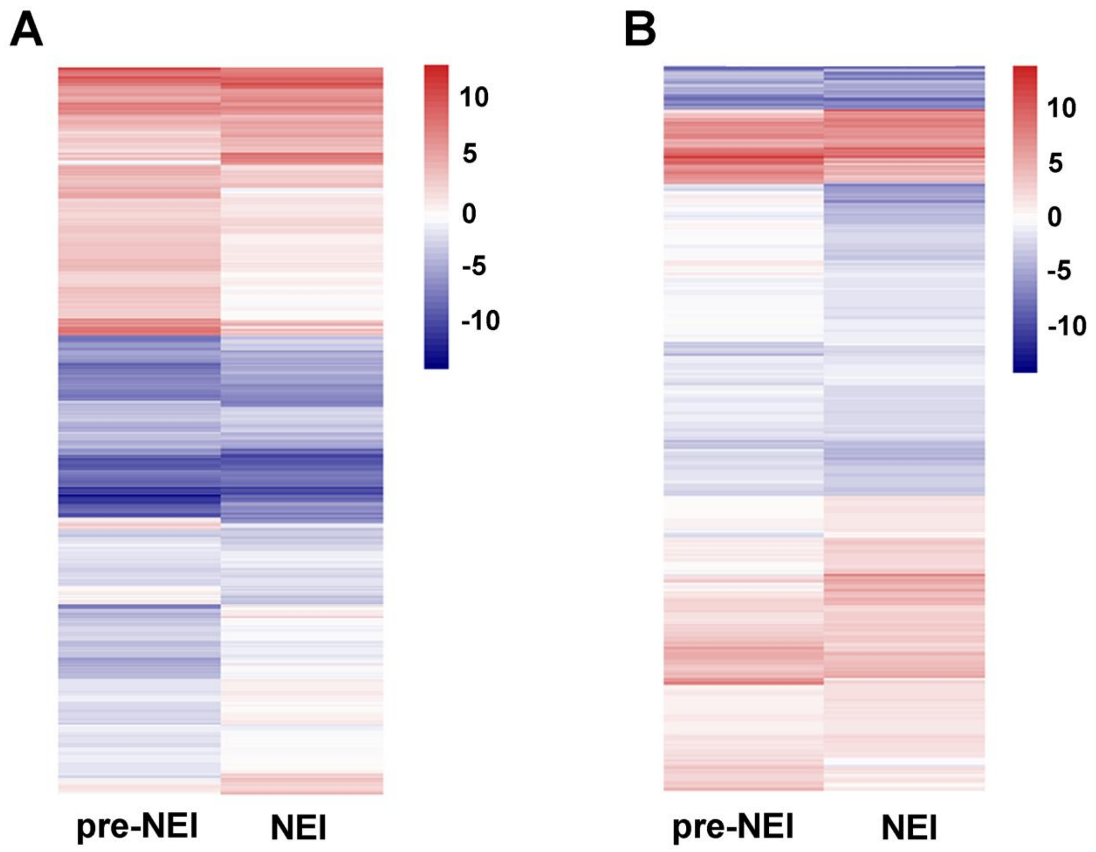

Figure S2 Heatmap of log2 transformed fold change of differentially expressed genes (DEGs) in the two pairs of PK/CM (A) and GF/PU (B). The fold change was calculated using edgeR package. PK: 'Huagan No.2' Ponkan (polyembryonic); CM: 'Nour' clementine (monoembryonic); GF: 'Cocktail' grapefruit (polyembryonic); PU: 'Huanong red' pummelo (monoembryonic). pre-NEI: stage right before the emergence of NEI cells; NEI: stage at the emergence of NEI cells.

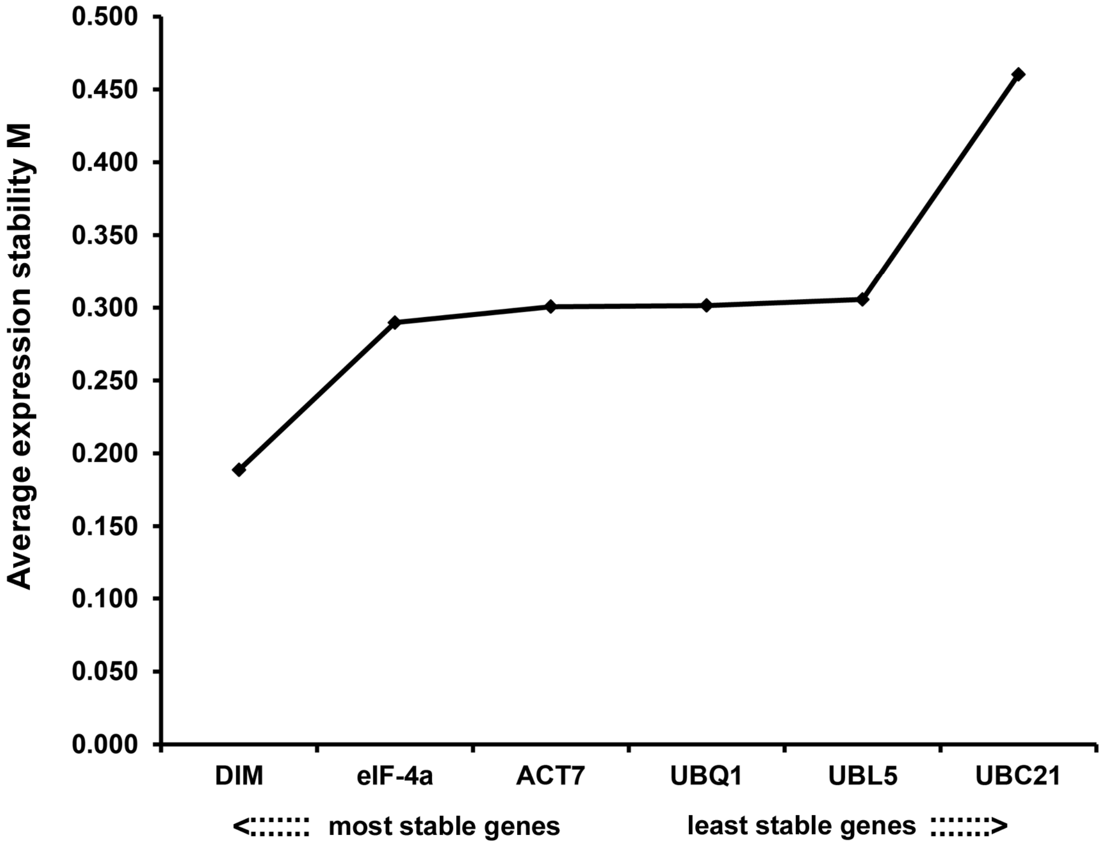

Figure S3 Selection of suitable reference genes for mRNA expression normalization. Gene-stability value *M* was calculated by geNorm for each candidate gene.

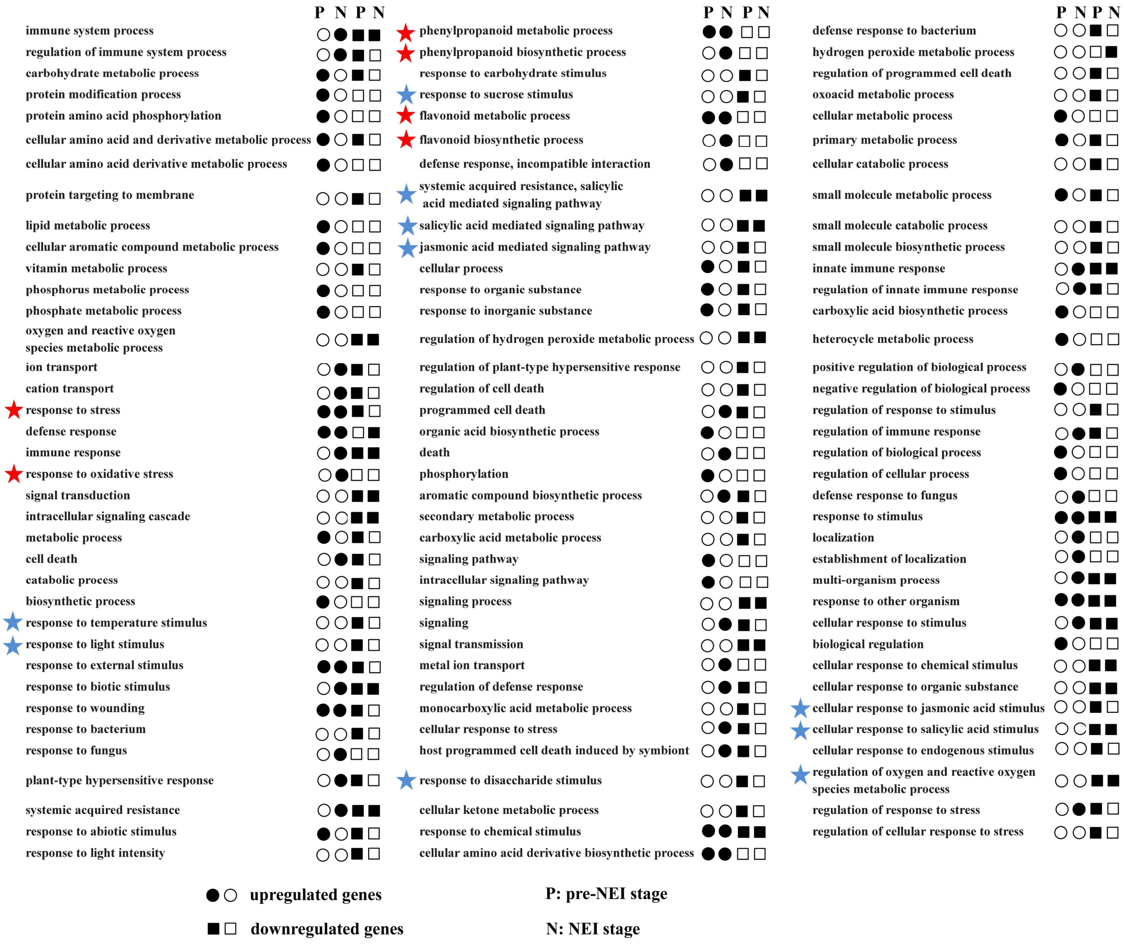

Figure S4 The overlapped enriched GO terms (biological processes) in PK/CM and GF/PU. GO enrichment analysis was performed based on the up-/downregulated genes at pre-NEI and NEI stage in PK/CM and GF/PU respectively, and the common enriched terms were extracted. The circle and quadrangle in black indicates the GO term enriched, whereas the hollow shapes indicate otherwise. The red and blue stars indicated that several biological processes were especially overrepresented based on the up- and downregulated genes respectively. pre-NEI: stage right before the emergence of NEI cells; NEI: stage at the emergence of NEI cells. PK: ‘Huagan No.2’ Ponkan (polyembryonic); CM: ‘Nour’ clementine (monoembryonic); GF: ‘Cocktail’ grapefruit (polyembryonic); PU: ‘Huanong red’ pummelo (monoembryonic).

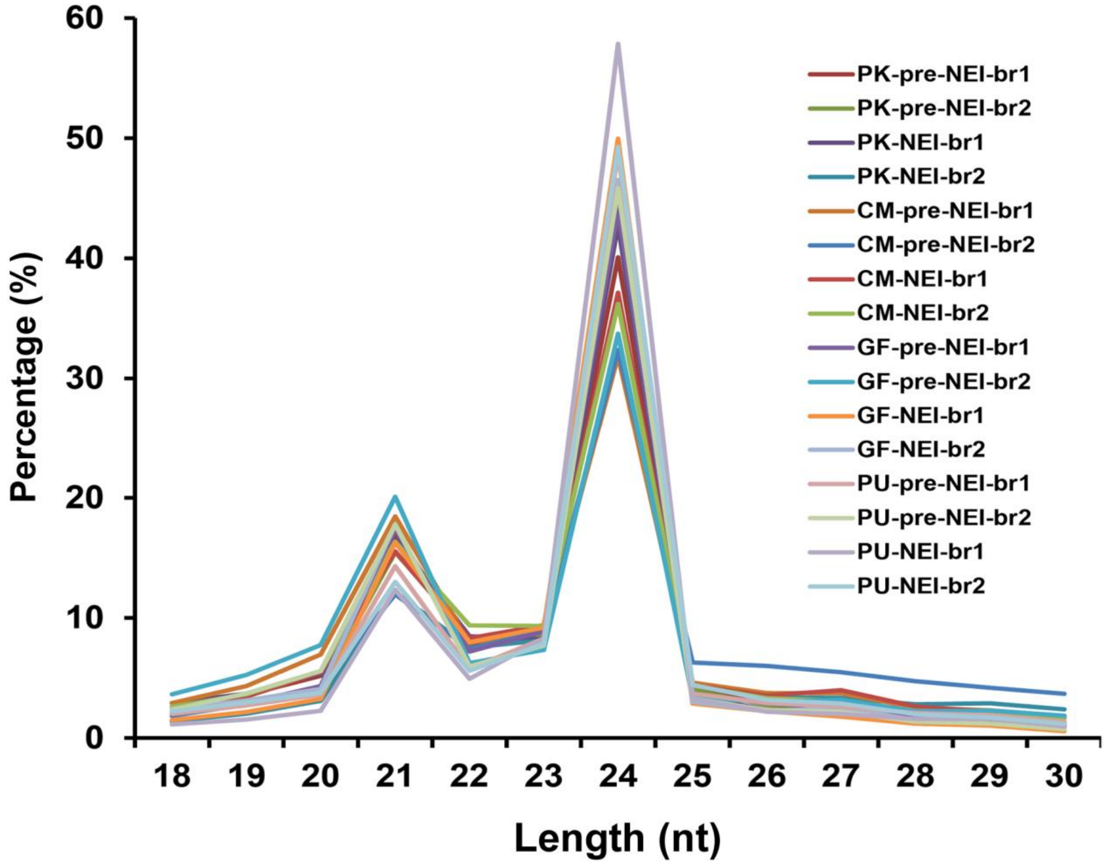

Figure S5 Length distribution of sRNA in 16 libraries. PK: 'Huagan No.2' Ponkan (polyembryonic); CM: 'Nour' clementine (monoembryonic); GF: 'Cocktail' grapefruit (polyembryonic); PU: 'Huanong red' pummelo (monoembryonic). pre-NEI: stage right before the emergence of NEI cells; NEI: stage at the emergence of NEI cells. br1/br2: two biological replicates respectively.

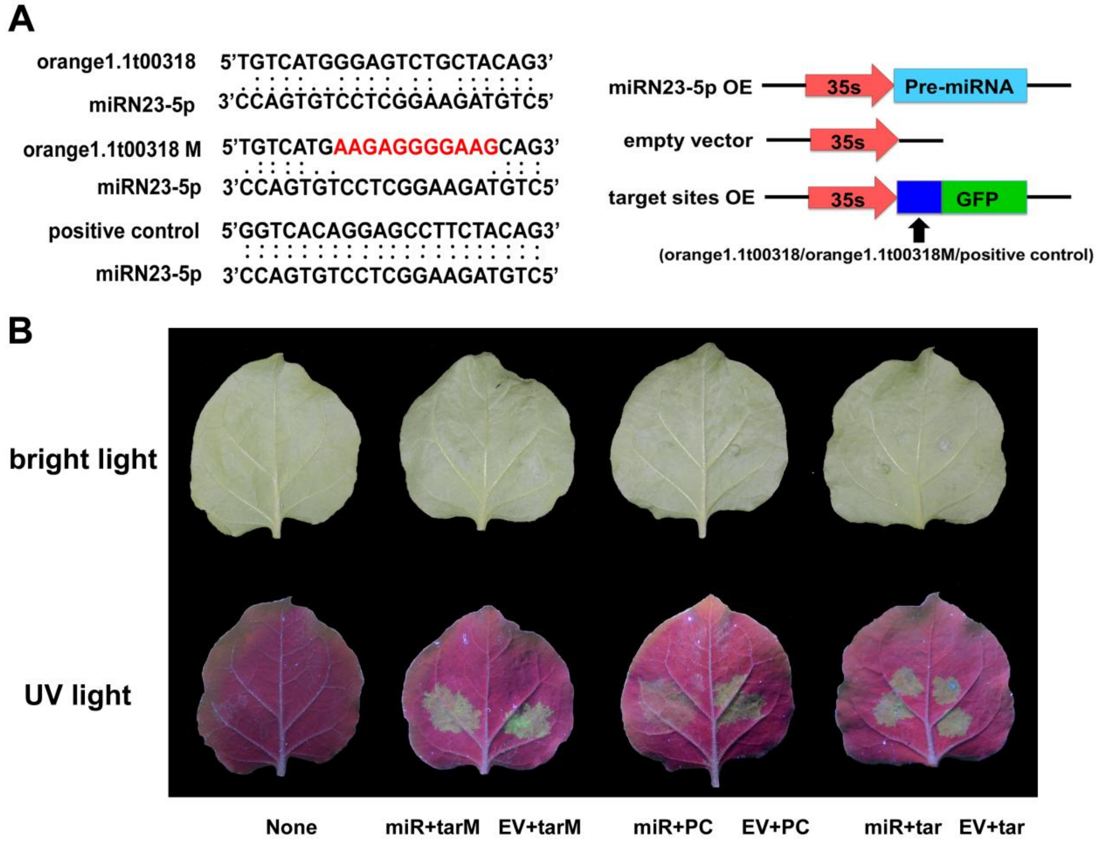

Figure S6 Transient assay in *Nicotiana benthamiana* could not validate miRN23-5p directed cleavage of the potential target orange1.1t00318. (A) Overexpression vectors were constructed for transient expression in *Nicotiana benthamiana*. miRN23-5p-OE: construct overexpressing miRN23-5p; empty vector as control; target sites-OE: construct overexpressing the native target sites (orange1.1t00318), the modified target sites insensitive to miRN23-5p (orange1.1t00318M); positive control: construct overexpressing target sites perfectly matched to miRN23-5p. (B) Co-infiltrated leaves and control were photographed at 3 days after infiltration under bright light (top) and UV light (bottom). All the leaves except the first one without any treatment were co-infiltrated with miRN23-5p (left) or empty control (right) along with native target Orange1.1t00318, modified target Orange1.1t00318M and positive control. None: no co-infiltration, negative control; miR: miRN23-5p, tar: native target Orange1.1t00318; tarM: modified target Orange1.1t00318M; PC: positive control.

Table S1 Primers used for qRT-PCR and transient assay

Gene name	primers
**mRNA detection**	
ACTL	**F**: CCAGGCAAGTCGCACAATAC **R**: GTGTTGGTTTGTGCCTCCCT
LAC	**F**: ACCTTCACGGCTACGAC **R**: GTGGGTTGACGAGATTG
AKN	**F**: GGTTCAAGTAAGACCCAAGGC **R**: CATTCAGTGTCTCCACCAGCA
CHI	**F**: ACCGACTCCTCAATATCCATG **R**: TCTCATCCAATACCACAAAGC
PHGPx	**F**: GCCTTTGCTCTGCTGTTGTC **R**: GGATGAAGTTGATGTGACGG
UGT	**F**: TTAAAACATGGCATGTCCCG **R**: AAGTTTCACTCCTTGGGTTGG
**miRNA detection**	
miR1446a	**RT**: GTCGTATCCAGTGCAGGGTCCGAGGTATTCGCACTGGATACGACCCATGA<br>**F**: TGCCGTTTCTAAACTCTCTCCC
miR393b-5p	**RT**: GTCGTATCCAGTGCAGGGTCCGAGGTATTCGCACTGGATACGACGATCAA<br>**F**:TTCATATTCCAAAGGGATCGCA
miR9560	**RT**: GTCGTATCCAGTGCAGGGTCCGAGGTATTCGCACTGGATACGACTTTCAT<br>**F**: TGATGTACAGGAGGTGGAACAAAT
miR827-3p	**RT**: GTCGTATCCAGTGCAGGGTCCGAGGTATTCGCACTGGATACGACTGTTTG<br>**F**: TGCCGCTTAGATGACCATCAA
miRN23-5p	**RT**: GTCGTATCCAGTGCAGGGTCCGAGGTATTCGCACTGGATACGACGGTCAC<br>**F**:TGCCGTCTGTAGAAGGCTCCT
miRN44	**RT**: GTCGTATCCAGTGCAGGGTCCGAGGTATTCGCACTGGATACGACCAGTTA<br>**F**: TGACGTAACGAGTCACTTTTCTGT
U6	**RT**: ACAGAGAAGATTAGCATGGCC<br>**F**: GACCAATTCTCGATTTGTGCG
Universal PCR reverse primer	GTGCAGGGTCCGAGGT
**miRNA target detection**	
Cs9g06920	**F**: GCAGCGTTCGGTCATACTTC **R**: TGCCGCTTCAGGACTAAAAT
orange1.1t00318	**F**: TGAAGAGCCTGCTCTACCAAC **R**: TCTTCACAACAGCCTGGTCC
**Transient assay**	
MIRN23-5p	**F**: AAAAAGCAGGCTCTTGAAAACAGTTATGCCTATTC<br>**R**: AGAAAGCTGGGTGTCTATTTTTTCAGCATGGAA
Cs9g06920	**F**: TCGAGCGTCACAACAGCCTTTTACGGT<br>**R**: CTAGACCGTAAAAGGCTGTTGTGACGC
Cs9g06920M	**F**: TCGAGCGTGGGGAAGAAAAGGGGCGGT<br>**R**: CTAGACCGCCCCTTTTCTTCCCCACGC
orange1.1t00318	**F**: TCGAGTGTCATGGGAGTCTGCTACAGT<br>**R**: CTAGACTGTAGCAGACTCCCATGACAC
orange1.1t00318M	**F**: TCGAGTGTCATGAAGAGGGGAAGCAGT<br>**R**: CTAGACTGCTTCCCCTCTTCATGACAC
positive control	**F**: TCGAGGGTCACAGGAGCCTTCTACAGT<br>**R**: CTAGACTGTAGAAGGCTCCTGTGACCC

Table S3 Summary of data statistics of sRNA libraries

Sample	Total Reads	Low Quality	Adaptor3 Null	Insert Null	5' Adaptor Contaminants	Size < 18 nt	PolyA	Clean reads	Genome matched (percentage)
PK-pre-NEI-br1	10,946,859	6,219	30,711	7,147	20,593	953,318	1,065	9,927,806	7,189,385(72.42%)
PK-pre-NEI-br2	13,179,184	20,752	68,363	13,281	24,981	790,872	2,385	12,258,550	9,583,145(78.18%)
PK-NEI-br1	12,696,266	8,225	36,061	133,332	27,311	1,409,546	1,900	11,079,891	7845563(70.81%)
PK-NEI-br2	15,049,725	26,569	77,869	30,384	33,255	716,469	2,860	14,162,319	11103736(78.4%)
CM-pre-NEI-br1	12,892,929	9,813	34,791	9,153	48,498	1,666,952	881	11,122,841	8527915(76.67%)
CM-pre-NEI-br2	10,162,054	15,090	44,095	17,255	19,208	540,089	681	9,525,636	8184887(85.92%)
CM-NEI-br1	13,971,190	8,504	45,083	45,936	28,747	1,126,760	1,675	12,714,485	9620901(75.67%)
CM-NEI-br2	10,849,566	18,620	46,593	27,358	26,607	975,376	1,053	9,753,959	8186513(83.93%)
GF-pre-NEI-br1	14,217,926	320,479	466,607	71,046	21,578	908,450	1,628	12,428,138	8575337(69.00%)
GF-pre-NEI-br2	12,332,377	8,024	40,051	6,547	54,474	2,322,489	757	9,900,035	6937613(70.08%)
GF-NEI-br1	12,972,466	259,164	379,121	42,390	16,743	688,219	1,378	11,585,451	7388474(63.77%)
GF-NEI-br2	13,901,096	7,794	40,973	9,819	56,348	1,693,586	1,204	12,091,372	7877499(65.15%)
PU-pre-NEI-br1	14,981,042	307,482	903,250	54,406	22,785	921,975	1,334	12,769,810	8455439(66.21%)
PU-pre-NEI-br2	12,758,370	7,203	48,915	13,991	39,792	1,110,797	1,053	11,536,619	7301389(63.29%)
PU-NEI-br1	17,246,675	322,453	688,965	79,910	24,159	686,036	2,218	15,442,934	9682784(62.7%)
PU-NEI-br2	17,296975	10,873	50,215	11,518	44,867	1,064,390	1,346	16,113,766	10177366(63.16%)

Table S4 Correlation coefficient between sRNA-seq biological replicates.

Sample	PK-pre-NEI-br1	PK-NEI-br1	CM-pre-NEI-br1	CM-NEI-br1	GF-pre-NEI-br1	GF-NEI-br1	PU-pre-NEI-br1	PU-NEI-br1
PK-pre-NEI-br2	0.94							
PK-NEI-br2		0.87						
CM-pre-NEI-br2			0.85					
CM-NEI-br2				0.87				
GF-pre-NEI-br2					0.82			
GF-NEI-br2						0.93		
PU-pre-NEI-br2							0.88	
PU-NEI-br2								0.94

Table S5 Identification and expression of known and novel miRNAs in the poly- and mono-embryonic ovules

Name	Sequence	Length	PU-pre-NEI-br1	PU-pre-NEI-br2	PU-NEI-br1	PU-NEI-br2	GF-pre-NEI-br1	GF-pre-NEI-br2	GF-NEI-br1	GF-NEI-br2	Precursor position
miR1446a	TTCTAAACTCTCTCCCTCATGG	22	4.1	3.2	3.5	2.6	2.6	0.7	0.1	1.3	chr2:11764129:11764284:+
miR1446b.1	GAACTCTCTCCCTCAACGGCT	21	5.9	2.3	7.1	0.8	5.0	2.2	9.0	4.8	chr7:15359605:15359749:-
miR1446b.2	CGAACTCTCTCCCTCAACGG	20	2.7	0.9	5.4	0.7	4.7	1.7	9.7	5.8	chr7:15359605:15359749:-
miR1515a	TCATTTTTGCGTGCAATGATCC	22	2.4	3.2	3.6	3.2	1.7	2.6	2.5	1.2	chr1:22740553:22740726:+
miR156a.1	TTGACAGAAGATAGAGAGCGC	21	0.0	1.2	0.0	2.1	0.0	0.2	0.0	0.3	chr2:10012388:10012490:+
miR156a.2	TGACAGAAGATAGAGAGCGC	20	0.2	0.3	0.3	0.1	0.0	0.1	0.3	0.3	chr2:10012389:10012489:+
miR156b.1	CTGACAGAAGAGAGTGAGCAC	21	0.5	0.3	0.8	0.3	0.2	0.4	0.8	0.2	chr4:392463:392578:+
miR156b.2	TGACAGAAGAGAGTGAGCAC	20	0.1	0.0	1.0	0.2	0.0	0.2	0.5	0.2	chr4:392463:392578:+<br>chr4:3430774:3430922:-
miR156c	ATGACAGAAGAGAGAGAGTAC	21	19.3	10.7	10.3	7.2	15.2	9.6	6.6	7.3	chr4:5740143:5740288:-
miR156d	TTGACGGAAGATAGAGAGCAC	21	7.6	8.1	24.9	11.9	7.7	4.6	15.6	6.4	chr6:18417834:18417934:-
miR156e	GTGACAGAAGATAGAGAGCGC	21	20.5	7.5	19.0	11.0	5.5	1.6	6.6	3.6	chr7:24974083:24974229:-
miR159	TTTGGATTGAAGGGAGCTCTA	21	6391.6	4802.7	6554.6	2858.9	6461.6	3263.2	7755.3	3735.7	chr5:31257481:31257730:-
miR160a	TGCCTGGCTCCCTGTATGCCG	21	2.5	1.6	1.6	1.5	2.5	0.6	1.9	1.8	chr2:7768976:7769118:+
miR160b	TGCCTGGCTCCCTGTATGCCA	21	84.5	47.2	41.6	41.6	59.3	30.5	44.7	40.7	chr7:28082699:28082840:-
miR162	TCGATAAACCTCTGCATCCAG	21	8.8	14.3	15.7	17.9	8.9	6.2	15.1	13.1	chrUn:57376063:57376213:+
miR164a	TGGAGAAGCAGGGCACGTGCA	21	23.5	11.1	22.1	6.8	10.6	3.3	9.9	3.9	chr3:18767350:18767491:+<br>chr5:34321631:34321781:-<br>chr6:11297780:11298009:+
miR166a	TCGGACCAGGCTTCATTCCCC	21	9582.1	11989.4	9405.8	12018.2	11557.2	6765.4	11176.9	13244.0	chr5:32144988:32145098:+<br>chr5:444253:444474:-<br>chrUn:6490895:6491092:+
miR166b	TCTCGGACCAGGCTTCATTCC	21	36081.1	29941.3	27992.1	22675.6	42071.1	27640.4	35098.0	31666.1	chr9:5710411:5710546:-<br>chr3:4882018:4882206:+
miR166c	TCGGACCAGGCTTCATTCCCT	21	3751.2	2969.5	3296.2	3365.2	4026.9	1965.9	3142.5	3425.9	chr3:4881893:4882008:+
miR166d	TTGGACCAGGCTTCATTCCTC	21	3.4	0.8	1.9	0.4	5.6	2.2	5.0	1.6	chrUn:22424158:22424369:+
miR166e	TCTTGGACCAGGCTTCATTCC	21	12.9	2.1	10.6	2.0	17.8	3.1	13.6	3.2	chrUn:22424313:22424412:+
miR167a.1	TGAAGCTGCCAGCATGATCTGA	22	14.8	17.1	16.6	15.9	7.7	6.8	8.6	9.1	chr2:8234557:8234897:+<br>chr3:14950351:14950486:+
miR167a.2	TGAAGCTGCCAGCATGATCT	20	19.3	18.7	13.3	7.7	25.9	14.2	26.7	17.9	chr3:14950351:14950486:+
miR167b-5p	TGAAGCTGCCAGCATGATCTTA	22	939.0	718.4	906.3	507.6	1001.4	378.8	1169.8	719.4	chr9:4850181:4850312:+
miR167b-3p	AGATCATGCGGCAGTTTCACC	21	404.7	341.1	310.2	651.9	319.8	151.4	208.3	330.0	chr9:4850181:4850312:+

miR168	TCGCTTGGTGCAGGTCGGGAA	21	74.9	72.6	136.4	82.6	81.1	56.5	102.8	75.4	ChrUn:55219628:55219825:+
miR169a.1	TAGCCAAGGATGACTTGCCT	20	2.6	1.6	2.8	2.4	7.2	1.8	9.5	7.3	chr1:911872:912035:+<br>chr2:10937954:10938108:+<br>chr2:10985273:10985404:+<br>chr2:10985564:10985718:+<br>chr2:11005804:11005921:+<br>chr2:11046802:11046954:+<br>chr2:11051077:11051288:+<br>chr2:11051229:11051387:+<br>chr2:27130623:27130801:+<br>chr2:27130919:27131029:+<br>chr6:14242833:14242963:-<br>chr7:1152858:1153060:-<br>chr7:9638905:9639035:-<br>chr7:16120763:16120880:-
miR169a.2	TAGCCAAGGATGACTTGCCTG	21	2.4	1.6	3.4	1.6	3.0	1.0	2.8	1.2	chr2:10937954:10938108:+<br>chr2:10985564:10985718:+<br>chr2:11005804:11005921:+<br>chr2:11046802:11046954:+<br>chr2:11051229:11051387:+<br>chr2:27130623:27130801:+<br>chr2:27130919:27131029:+
miR169a-3p	AGGCAGTCTCCTTGGCTAAC	20	2.0	2.9	2.8	1.9	0.1	0.3	0.6	0.2	chr2:27130623:27130801:+
miR169b	CAGCCAAGGATGACTTGCCGG	21	0.1	0.2	0.6	0.1	0.2	0.1	0.1	0.2	chr6:20613054:20613191:+<br>chr7:4557015:4557100:-<br>chr9:1732724:1732859:+
miR169c	TAGCCAAGGATGACTTGCCTGCG	23	0.2	0.2	0.2	0.0	0.0	0.0	0.3	0.0	chr2:11046796:11046934:+
miR171a-3p	TGATTGAGCCGTGCCAATATC	21	53.2	47.9	36.7	30.1	50.1	19.8	27.8	20.5	chr1:28276647:28276804:+<br>chr2:18394832:18394956:+
miR171a-5p	TATTGGCCTGGTTCACTCAGA	21	16.4	28.0	11.2	9.1	10.7	11.3	8.2	7.9	chr1:28276647:28276804:+
miR171b	TTGAGCCGTGCCAATATCACG	21	0.3	0.0	0.0	0.0	3.2	1.3	2.9	2.6	chr2:1312568:1312672:+
miR171c	TATTGGCCCGGTTCATTCAGA	21	24.3	18.0	47.1	13.8	16.0	7.0	19.9	8.9	chr3:27931456:27931594:-
miR171d	TGAATGAACCGGGCCAATATC	21	0.5	0.4	0.2	0.3	0.1	0.4	0.1	0.7	chr3:27931477:27931576:+
miR171e-3p	TTGAGCCGCGCCAATATCACT	21	1.4	0.6	1.6	1.2	1.0	0.4	0.9	0.6	chr4:3390372:3390517:-
miR171e-5p	CGATATTGGTGAGGTTCAATC	21	0.6	0.3	1.5	0.7	0.7	2.1	1.5	0.0	chr4:3390372:3390517:-
miR171f	TTGAGCCGCGTCAATATCTCC	21	1.5	1.1	1.9	0.7	1.7	0.8	2.5	1.3	chr2:18268124:18268360:+
miR171g	CGAGCCGAATCAATATCACTC	21	0.3	1.3	1.7	0.7	2.0	0.8	2.8	3.2	chrUn:20985561:20985718:+
miR172a	GCAGCGTCCTCAAGATTCACA	21	129.3	111.1	64.8	22.1	86.9	68.4	37.5	25.1	chr1:27308841:27309005:-
miR172b.1	AGAATCTTGATGATGCTGCA	20	3.4	1.6	2.7	0.1	4.5	1.7	2.8	0.7	chr7:24626933:24627122:+<br>chr8:19105385:19105490:+
miR172b.2	AGAATCTTGATGATGCTGCAT	21	4.5	3.0	1.5	0.3	2.0	1.4	2.3	1.3	chr7:24626933:24627122:+<br>chr8:19105366:19105511:+

miR2111	TAATCTGCATCCTGAGGTTTG	21	0.3	0.0	0.3	0.0	0.2	0.0	0.2	0.0	chr6:8975741:8975828:-
miR2275a	TTTAATTTCCTCCAATATTTCA	22	0.3	0.4	0.2	0.2	0.1	0.0	0.0	0.0	chr1:15317257:15317387
miR2275b	TTTAGTTTCCTCCAATATCTTA	22	0.7	0.9	0.4	0.4	0.7	0.4	0.4	0.6	chr6:20310988:20311120:+<br>chrUn:49594681:49594824:+
miR319a.1	CTTGGACTGAAGGGAGCTCCT	21	63.8	30.6	41.1	15.8	24.0	19.4	25.7	23.0	chr1:9887405:9887599:-<br>chr7:29667408:29667602:-
miR319a.2	TTGGACTGAAGGGAGCTCCT	20	54.3	20.4	41.8	11.6	27.6	21.4	31.0	25.1	chr1:9887405:9887599:-<br>chr7:27372746:27372939:+
miR319b	TTTGGACTGAAGGGAGCTCCT	21	1047.2	754.9	1209.1	797.3	1315.7	1129.0	1636.1	1649.0	chr4:17297190:17297283:+
miR319c	TTGGACTGAAGGGAGCTCCC	20	13.5	6.4	8.5	2.3	16.3	10.6	14.6	10.9	chr5:1270908:1271094:+<br>chr2:10333391:10333581:+
miR319d	ATCCAACGAAGCAGGAGCTGC	21	0.5	0.6	0.6	0.3	0.9	0.6	0.8	0.5	chr2:10333433:10333539:+
miR390a	AAGCTCAGGAGGGATAGCGCC	21	244.0	195.4	251.9	116.0	258.5	130.4	290.0	156.3	chr6:11042424:11042582:+<br>chr8:22526276:22526434:+
miR393a.1	TCCAAAGGGATCGCATTGATCC	22	2.7	1.6	6.0	2.5	2.7	1.7	6.0	3.1	chr2:28762177:28762277:-<br>chr7:31958695:31958837:+
miR393a.2	TCCAAAGGGATCGCATTGATC	21	3.8	2.9	4.5	2.2	3.5	1.2	4.6	1.2	chr2:28762177:28762277:-<br>chr7:31958695:31958837:+
miR393b-3p	TCATGCGATCCCTTCGGAATT	21	39.5	30.3	29.3	26.1	12.3	6.0	7.9	10.3	chr9:16703325:16703498:-
miR393b-5p	TTCCAAAGGGATCGCATTGATC	22	68.6	54.4	73.0	37.7	19.5	9.3	19.6	12.6	chr9:16703325:16703498:-
miR394a	TTGGCATTCTGTCCACCTCC	20	95.9	59.0	124.0	52.3	91.0	33.1	99.3	46.1	chr3:27990508:27990679:+
miR3951-5p	TAGATAAAGATGAGAGAAAAA	21	74.4	82.7	107.0	46.7	50.6	20.5	93.0	44.4	chr4:7897900:7898068:-
miR3951-3p	TTTCTCTTATCGTTATCTGTG	21	0.5	0.3	0.5	0.2	63.9	49.4	61.5	48.1	chr4:7897900:7898068:-
miR3952	TGAAGGGCCTTTCTAGAGCAC	21	1010.0	956.8	1488.0	736.9	1031.9	711.5	1334.6	949.3	chr8:15996320:15996520:+<br>chr8:16164233:16164395:+
miR3954	TTGGACAGAGAAATCACGGTCA	22	381.4	273.0	338.1	189.3	346.6	153.5	313.6	155.1	chr9:5824568:5824743:-
miR396a	TTCCACAGCTTTCTTGAACTT	21	117.2	124.6	98.8	64.7	104.2	79.2	82.1	90.1	chr1:15269146:15269349:-<br>chr4:3340888:3341059:-
miR396b-5p	TTCCACGGCTTTCTTGAACGTA	22	0.1	0.0	0.1	0.2	0.3	0.0	0.2	0.1	chr1:28388120:28388277:+
miR396b-3p	CGTTCAAGAAGCTGTGGAAAA	21	0.1	0.2	0.1	0.4	0.1	0.1	0.0	0.2	chr1:28388120:28388277:+
miR396c	TTCCACAGCTTTCTTGAACTG	21	10.5	1.5	9.6	2.0	12.9	2.3	13.6	3.9	chr4:3335310:3335457:+<br>chr7:3041253:3041360:-
miR396d	TTCCACGGCTTTCTTGAACTT	21	18.0	27.4	15.7	26.3	14.0	14.1	18.0	39.0	chr7:10781005:10781174:-
miR397a	TCATTGAGTGCAGCGTTGATG	21	2.4	1.1	3.8	2.4	1.9	2.5	2.6	9.4	chr2:4101109:4101221:-
miR398	TGTGTTCTCAGGTCACCCCTT	21	6.7	4.7	20.0	23.5	0.6	0.5	0.7	0.4	chr7:1333375:1333571:+
miR399a	CGCCAAAGGAGAATTGCCCTG	21	0.0	0.1	0.0	0.2	0.6	0.2	3.1	2.6	chr2:2298416:2298548:-
miR399b	TGCCAAAGGAGAATTGCCCTG	21	0.0	0.1	0.0	0.2	0.3	0.0	0.9	0.9	chr2:2298415:2298547:+
miR399c	TGCCAAAGGAGATTTGCCCGG	21	0.0	0.1	0.0	0.0	0.9	0.1	0.9	0.6	chr2:2305455:2305548:+
miR399d	TGCCAAAGGAGAGTTGCCCTA	21	0.5	0.5	0.6	1.0	1.4	1.4	3.1	3.9	chr5:7022860:7023017:-

miR403	TTAGATTCACGCACAAACTCG	21	507.1	511.3	641.0	438.1	365.3	193.8	457.3	322.2	chr9:719780:719884:+<br>chrUn:27643336:27643465:-
miR472	TTTTTCCCACACCTCCCATCCC	22	516.4	419.0	509.2	427.2	364.7	189.2	447.5	371.8	chr2:9962406:9962536:+
miR473	ACTCTCCCTCAAGGGCTTCGC	21	2.5	6.3	7.6	3.9	7.2	4.8	24.6	16.6	chr7:9419844:9419986:+
miR477a	ACTCTCCCTCAAGGGCTTCTGA	22	2.3	3.2	1.8	1.1	2.7	1.4	5.8	1.9	chr5:1063936:1064126:-
miR477b	ACTCTCCCTCAAGGGCTTCTGG	22	1.0	1.3	0.6	0.4	0.4	0.7	1.5	0.2	chr5:1064064:1064212:-
miR477c	ACTCTCCCTCAAGGGCTTCTC	21	0.6	2.0	1.9	0.9	3.0	3.0	7.7	6.8	chr7:9419646:9419801:+
miR479	TGTGATATTGGTTCGGCTCATC	22	1.5	1.7	2.0	1.1	2.8	1.0	8.6	3.1	chrUn:20985561:20985718:+
miR482a	TTGCCAACTCCTCCCATGCCGA	22	1704.1	1503.5	1846.6	1206.0	1981.2	977.1	1642.1	1586.8	chr2:9926618:9926743:+
miR482b	TCTTGCCCACCCCTCCCATTCC	22	140.8	227.4	171.5	210.6	138.9	118.1	171.5	234.8	chr2:9932099:9932244:+
miR482c	TCTTGCCAAGCCTCCCATTCC	21	0.4	0.5	0.5	0.3	0.2	0.1	0.1	0.2	chr2:9932117:9932225:-
miR482d	TCTTCCCTATGCCTCCCATTCC	22	604.6	500.0	464.0	458.1	547.1	297.2	492.4	474.8	chr2:9940988:9941153:+
miR482e	TCTTACCTATGCCACCCATTCC	22	1535.4	1569.2	988.6	1096.8	1589.0	899.7	1026.2	956.7	chr2:9946465:9946595:+
miR482f	TCCCTACTCCACCCATGCCATA	22	362.9	398.0	366.4	288.9	249.4	215.0	270.7	375.6	chr6:1186339:1186511:+
miR482g	TTCCCTAGTCCCCCTATTCCTA	22	218.4	219.4	214.9	199.3	261.3	145.4	222.9	260.5	chr2:9926820:9926957:+
miR5179	TCTTGCTCAAGACCGCGCAAT	21	3.7	0.3	6.1	1.1	4.4	1.5	7.4	2.6	chr5:12798100:12798317:+
miR530	TGCATTTGCACCTGCACCTTG	21	3.9	5.1	2.5	1.2	1.4	0.7	1.3	0.9	chr8:18991475:18991598:+
miR535	TGACAATGAGAGAGAGCACAC	21	0.8	0.7	0.5	0.4	0.2	1.1	1.5	1.2	chr2:14867757:14867897:-
miR827-5p	CTTGTTGATTGTCATCTAATC	21	67.3	84.0	214.4	136.0	92.5	82.5	229.2	183.8	chrUn:38140507:38140646:+
miR827-3p	TTAGATGACCATCAACAAACA	21	35.2	47.3	54.2	61.0	31.4	20.2	41.9	40.4	chrUn:38140507:38140646:+
miR828	TCTTGCCTAAATGAGTATCCC	21	0.2	0.6	0.1	0.4	0.0	0.0	0.0	0.0	chrUn:6263210:6263358:-
miR9560	ACAGGAGGTGGAACAAATATGAAA	24	0.3	2.2	5.9	32.3	0.7	0.6	0.3	0.4	chr3:18989004:18989087:-
**miRN01**	TAGAACCTTTTAATTAATAAA	21	8.0	2.1	11.4	1.7	8.6	0.4	15.6	0.9	chr1:1908382:1908540:+
miRN02-5p	AAACCGAACCGAATTTTAGAACCG	24	0.8	0.4	0.6	0.3	22.9	14.7	33.8	29.8	chr1:5988673:5988808:-
miRN02-3p	TTCTAAAATTCGGTTCGGTTT	21	3.7	6.1	2.3	4.0	49.6	38.4	40.4	50.4	chr1:5988673:5988808:-
**miRN03**	GCTCTAAAGTGTTTGGTAAACACT	24	2.0	3.0	1.7	4.7	3.8	4.6	2.1	3.9	chr1:15621784:15621990:-
miRN04	TACAGTCGTTTCATGAAAATC	21	4.9	7.1	9.8	4.6	2.3	1.7	4.0	4.1	chr2:4879288:4879444:-
**miRN05**	TGTTTTGGGTGAAACGGGTGTT	22	21.7	28.9	28.6	18.3	44.4	28.5	37.4	24.8	chr3:13420801:13420953:-<br>chrUn:33972455:33972607:+
miRN06-3p	TCCCACCACCTTGGCATGCTGAGG	24	15.3	10.8	15.0	10.0	5.1	6.0	8.5	5.3	chr5:8697314:8697466:-
miRN06-5p	TCAACATGTCGAAGTGGTGGGATA	24	6.5	6.7	7.4	5.5	4.3	1.4	3.1	1.7	chr5:8697314:8697466:-
miRN07	TAGTGAATTCTGTCTATTTGGATA	24	0.7	0.0	0.0	0.0	66.6	9.7	81.6	18.9	chr6:16245472:16245668:+
**miRN08**	CCGTTTCATCTTGTCCTCCAG	21	187.4	198.0	130.7	124.2	275.9	172.7	230.8	244.4	chr8:8020103:8020210:+
miRN09	TGAAGGATGTATGTTACAATT	21	7.9	10.2	7.3	5.0	0.2	0.1	0.3	0.2	chr8:11115878:11115986:-
**miRN10**	ACCGTAGGTGAACTCTAACATAGC	24	0.8	0.2	0.1	0.2	54.6	42.0	84.4	69.2	chrUn:4101718:4101931:+
**miRN11**	AGTTTCAGGATTGGTTTGGGATTC	24	14.0	16.7	9.9	12.0	18.7	11.8	17.7	16.4	chrUn:5774355:5774540:+
miRN12	AACATAATTGGGGAATAGATTCTC	24	0.2	0.0	0.1	0.0	5.0	3.9	6.0	7.0	chrUn:46845059:46845236:+

**miRN13**	ATTTTAAAACGTGCGGATGTCTGC	24	39.5	23.4	53.1	20.5	90.7	35.2	95.4	34.7	chrUn:51273878:51274071:+
miRN14-5p	TGACAATGTTACTCACCATAAAGT	24	33.9	53.5	48.9	44.6	18.6	8.8	30.3	15.5	chrUn:60037188:60037313:+
**miRN14-3p**	ACGTTATGGTGAGCGACACTGTCA	24	4.8	11.4	5.6	9.9	7.2	11.0	7.8	17.7	chrUn:60037188:60037313:+
miRN15	AATGCCCTTAAAACAATCCAACCA	24	1.5	3.7	4.2	4.3	1.4	2.8	2.2	4.1	chr2:6386280:6386559:-
miRN16	CAGACTCTGTATGTAACGCAGC	22	4.5	4.9	6.4	3.8	0.0	0.0	0.1	0.1	chr2:7457049:7457230:-
miRN17	AATTCTAATTGTAATTACACTGCC	24	1.2	2.0	1.5	0.9	10.0	5.4	10.4	7.7	chr2:26933562:26933759:-
miRN18	TGAGTCATGGATCTAATGCAA	21	8.3	3.3	7.3	2.2	4.5	1.2	4.4	1.4	chr3:15820273:15820448:-
miRN19	TTCATATTTCATATTTACATCACA	24	5.5	4.8	5.6	5.3	7.5	4.1	4.1	3.1	chr5:27297871:27298261:+
miRN20	CCAATTGGGATCCCGCACTTTAGT	24	0.0	0.0	0.1	0.0	3.1	2.9	4.0	7.1	chr6:17770069:17770252:-
miRN21	TTGTGGGTTGTGTATGTTACA	21	7.1	6.7	3.7	4.6	3.9	2.9	2.7	3.6	chr7:10179965:10180104:-
miRN22	AGGGATGTACCATACATTGATTGT	24	7.6	4.2	11.9	3.5	2.3	0.7	2.2	1.0	chr8:8851005:8851117:-
**miRN23-3p**	TCGCAGGAGCTTTCTACGGTT	21	220.4	175.0	230.9	105.7	67.6	29.0	51.5	34.8	chr8:16330188:16330404:+
**miRN23-5p**	CTGTAGAAGGCTCCTGTGACC	21	119.2	124.4	154.2	74.2	37.6	24.8	39.6	23.2	chr8:16330188:16330404:+
miRN24	ATCCAACTGTGGTACCATACAACT	24	10.8	8.9	11.0	7.0	3.1	1.7	4.8	2.8	chr9:1308595:1308716:+
**miRN25**	CGTCCCCCTCACGGCTACAGTACC	24	0.4	0.0	0.1	0.0	36.2	56.8	56.3	65.8	chrUn:4098056:4098137:+
**miRN26**	TAAAATATTGAGTGAGAGAGGAAA	24	2.2	2.1	5.1	3.2	2.8	0.6	1.7	1.1	chrUn:8736640:8736764:+
miRN27	AGAGGTTGTATGGCTTAGAAAGTT	24	127.0	155.2	120.7	107.6	116.8	82.4	105.4	94.8	chrUn:14144498:14144825:-
miRN28	TAAAGAATTCTTTACACGACTGTT	24	4.2	3.3	4.3	3.2	1.0	0.6	0.9	0.5	chrUn:36109386:36109469:-
miRN29	TATACGTCACTGTTCATGTATACT	24	2.4	1.8	1.7	1.4	5.0	2.5	4.1	3.8	chr8:27186:27434:+
miRN30	TATGTTTGATCAAGCTGCTTTTCC	24	8.5	4.0	4.5	1.0	3.7	1.7	4.1	2.3	chr4:19505019:19505229:-
miRN31	ATGAGTAAGTGGGAGGCATATGGT	24	15.8	9.5	15.5	5.7	49.8	14.3	44.5	15.5	chr9:3940907:3941116:-
**miRN32**	ATTGGTCGGGTGCACCACCTCAGC	24	160.2	171.0	210.5	152.4	167.4	141.8	196.5	151.0	chr8:7523219:7523466:+
**miRN33**	TCCTTAGTTGAGTTGGTTGGCATT	24	26.5	53.0	34.8	51.9	21.6	22.3	24.8	34.6	chrUn:5431384:5431571:-<br>chr6:10846775:10846962:+
miRN34-5p	TTATCCATTCATTCCTTGCCATGT	24	28.0	38.1	21.0	14.8	32.8	17.9	25.6	17.9	chr7:26424637:26424808:+
miRN34-3p	GTGGCAAATAGTGAATGGATAAGG	24	18.9	20.0	22.0	14.6	20.8	14.2	23.4	13.7	chr7:26424637:26424808:+
miRN35	CGGGATTGAGACATATTTTTATCC	24	0.0	0.0	0.1	0.0	3.9	0.9	12.4	4.5	chr1:8829372:8829618:-
**miRN36**	AAATCACTAACCGTCAAGTATAAG	24	23.3	30.8	34.7	29.7	11.7	7.3	13.8	10.6	chr2:16521115:16521354:+
miRN37	TAATTAAGTTGAAATTAGTAGACT	24	18.1	19.9	59.7	69.6	0.0	0.0	0.0	0.0	chr1:13034319:13034567:-
miRN38	TGTCAATTGGTCCAGGATTTTAAT	24	3.4	3.9	1.8	1.1	4.6	2.5	2.8	2.2	chr5:4414799:4414924:-
miRN39	CACAGTGTAGTATACATGAACAAC	24	0.9	1.1	0.7	0.9	5.0	3.5	3.7	6.8	chr1:19232:19473:-
miRN40	TTTGCTTTTGAAGATTATCAGGAG	24	81.8	62.2	151.5	89.1	58.3	40.3	86.1	78.4	chrUn:66804147:66804394:+<br>chrUn:66805140:66805387:+
miRN41	ACCGTTGCACCCAGCCGTTGGATC	24	3.3	25.7	1.5	0.9	2.5	3.9	1.1	3.9	chr4:6654503:6654636:-
miRN42	CGGCTTTGCTCCACGAATCAT	21	82.9	77.1	95.2	78.3	87.1	41.8	117.4	73.4	chr1:12278151:12278393:+
miRN43	ATGGACTACTTCTAGAATGGT	21	23.2	28.5	15.9	10.4	13.2	8.2	5.2	4.5	chr2:9445857:9446056:-
**miRN44**	AACGAGTCACTTTTCTGTTAACTG	24	0.1	0.2	0.1	0.0	34.8	37.7	28.2	31.6	chr2:29993661:29993907:-

miRN45	ATGAGAAAGGTTTATGATGTTGAG	24	9.9	8.1	22.8	11.2	8.2	6.9	14.2	7.1	chrUn:48466074:48466321:+
**miRN46**	AACTTACACCAACTCACATGAACT	24	9.5	12.2	7.1	10.7	13.8	8.0	13.6	12.2	chr5:28153678:28153871:-
miRN47	GACTGTCCCATCTAAGTTTTTCTC	24	9.9	7.5	11.6	8.8	8.0	5.7	11.8	6.1	chr5:8697516:8697648:-
miRN48-3p	CTTTTCATGATTTCCTGTGGCACA	24	12.2	18.5	13.1	13.8	5.6	7.5	7.5	6.5	chr9:3942293:3942512:-
miRN48-5p	TGCTTCAAAAAATCGTGGAAAGTA	24	11.5	11.1	12.6	11.9	3.9	14.8	2.8	21.4	chr9:3942293:3942512:-
miRN49	AACGTCGTAAACTCGTCTCGTACT	24	4.2	4.9	4.1	5.0	11.9	6.9	14.7	13.6	chr3:13535449:13535633:+
miRN50	GATAATAAGAATTCTGATGGTACC	24	128.7	57.9	73.1	39.7	0.2	0.0	0.3	0.0	chr2:12898092:12898326:-
miRN51	ATAAGAAAAATTGAACTTGGCAGC	24	3.1	0.0	0.8	0.1	135.1	61.3	311.7	69.1	chr4:4090193:4090440:+
miRN52	TTTTGTTGCATGATGCTGATAA	22	8.5	7.8	10.4	10.0	13.6	9.8	14.3	15.2	chr4:7394616:7394726:-
miRN53	CTTTCAGCAGCCTCCGGCGTC	21	16.1	31.8	19.1	20.7	16.1	31.8	16.4	23.0	chr6:12585665:12585808:+<br>chr6:12587579:12587722:+
miRN54	TCTCAGGTCGCCCCTGTGGGA	21	0.9	1.2	2.2	1.9	0.6	1.6	2.8	19.5	chr2:28955938:28956087:+
**miRN55**	TATACGTCGTTGTTCATGTATACT	24	18.7	17.4	18.5	15.0	70.9	44.1	67.4	70.0	chr1:19242:19483:+<br>chr1:23322:23563:+
**miRN56**	ATCTATTTGGATGAAGATAAGGGC	24	24.0	19.2	19.6	30.7	27.6	34.6	24.8	36.9	chr7:31634781:31635013:-
**miRN57**	ATAAAAAACACTCTCAAACGCACC	24	474.0	368.8	692.4	469.5	508.0	356.3	669.9	598.3	chr1:26922935:26923093:+
miRN58	AGTATGCAGAATCTCAACGTCTTG	24	12.3	10.3	16.0	11.9	7.4	3.0	9.8	9.3	chrUn:48466074:48466321:+
miRN59	GACGGGATTGAGACATATTTTTAT	24	0.1	0.0	0.0	0.0	4.1	2.8	10.8	12.2	chr1:8829372:8829618:-
miRN60	TTGTCGCCGGAGAGATAGCACC	22	16.6	24.9	30.2	15.3	24.5	15.1	23.7	20.3	chr6:19944523:19944750:-

Name	Sequence	Length	CM-pre-NEI-br1	CM-pre-NEI-br2	CM-NEI-br1	CM-NEI-br2	PK-pre-NEI-br1	PK-pre-NEI-br2	PK-NEI-br1	PK-NEI-br2	Precursor position
miR1446a	TTCTAAACTCTCTCCCTCAAGG	22	4.4	2.2	3.0	4.3	0.0	0.0	0.1	0.0	scaffold_2:11672871:11673026:-
miR1446b.2	CGAACTCTCTCCCTCAACGG	20	1.4	1.2	2.9	5.3	3.7	0.8	3.5	0.7	scaffold_4:14417461:14417564:+
miR1515	TCATTTTTGCGTGCAATGATCC	22	1.6	0.4	0.9	1.3	2.3	1.6	2.3	2.0	scaffold_7:5838587:5838761:-
miR156a.1	TTGACAGAAGATAGAGAGCGC	21	0.2	0.0	0.4	0.0	0.7	0.0	0.4	0.3	scaffold_2:28474072:28474174:-
miR156b.1	CTGACAGAAGAGAGTGAGCAC	21	0.4	0.3	0.3	0.5	0.6	0.6	0.9	0.4	scaffold_1:28593463:28593577:-
miR156b.2	TGACAGAAGAGAGTGAGCAC	20	0.4	0.2	1.3	0.3	0.0	1.5	0.0	0.1	scaffold_1:1110815:1110915:-<br>scaffold_1:25708441:25708546:+<br>scaffold_1:28593463:28593577:-
miR156b.3	TGACAGAAGAGAGTGAGCACA	21	0.1	0.2	0.0	0.0	0.0	0.2	0.1	0.6	scaffold_1:1110815:1110915:-<br>scaffold_1:25708441:25708546:+<br>scaffold_1:28593463:28593577:-
miR156c	ATGACAGAAGAGAGAGAGTAC	21	2.7	9.4	5.9	15.1	15.0	35.9	19.1	15.3	scaffold_1:23475302:23475447:+
miR156d	TTGACGGAAGATAGAGAGCAC	21	9.5	6.2	16.9	11.6	18.7	12.6	35.3	14.0	scaffold_6:22903916:22904057:-
miR156e	GTGACAGAAGATAGAGAGCGC	21	1.8	3.5	2.4	7.5	3.7	10.4	3.8	8.1	scaffold_1:15209049:15209195:-
miR159	TTTGGATTGAAGGGAGCTCTA	21	2386.1	1026.5	3923.5	2513.1	4260.5	2478.9	5784.5	2009.3	scaffold_3:42897060:42897309:-

miR160a-3p	GCGTACGAGGAGCCAAGCATA	21	0.1	0.5	0.0	1.9	0.1	2.4	0.0	1.1		scaffold_2:30834767:30834909:-
miR160a-5p	TGCCTGGCTCCCTGTATGCCG	21	1.1	0.4	1.0	1.9	2.6	1.5	1.7	1.1		scaffold_2:30834767:30834909:-
miR160b	TGCCTGGCTCCCTGTATGCCA	21	17.2	10.2	21.9	42.0	44.1	51.9	25.9	39.3		scaffold_4:3991028:3991169:+
miR162	TCGATAAACCTCTGCATCCAG	21	9.9	6.2	26.4	15.2	8.1	16.8	53.0	21.3		scaffold_3:8951064:8951214:-
miR164a-3p	CACGCGCTCCCCTTCTCCAAC	21	4.0	2.0	4.4	3.2	4.0	4.2	7.3	3.0		scaffold_5:33408122:33408263:+
miR164a-5p	TGGAGAAGCAGGGCACGTGCA	21	1.3	0.6	2.0	2.7	2.3	3.7	2.2	2.0		scaffold_5:33408122:33408263:+
miR164b	CATGTGCCCTAGCTCTCCAGC	21	0.5	0.2	0.6	0.2	0.6	0.2	0.1	0.1		scaffold_7:7665831:7665927:+
miR166a	TCGGACCAGGCTTCATTCCCC	21	5119.6	5642.8	4022.2	12632.6	7426.7	16376.9	6909.0	12232.6		scaffold_3:43731635:43731745:+<br>scaffold_3:439810:440031:-
miR166b	TCTCGGACCAGGCTTCATTCC	21	32174.1	23607.8	18921.9	29017.4	30267.0	28733.5	16344.0	16420.2		scaffold_3:47795048:47795245:-<br>scaffold_9:4741023:4741158:-<br>scaffold_3:13055746:13055875:+<br>scaffold_5:9961644:9961826:-
miR166c	TCGGACCAGGCTTCATTCCCT	21	788.1	520.0	1062.3	1344.2	1870.5	1693.6	1985.2	1450.9		scaffold_5:9961817:9961969:-
miR166d	TTGGACCAGGCTTCATTCCTC	21	20.9	32.3	23.4	44.4	133.1	131.1	157.5	74.1		scaffold_7:15646319:15646481:+<br>scaffold_7:15646401:15646637:+
miR166e	TTGGACCAGGCTTCATTCCAC	21	5.2	9.3	5.0	6.3	57.5	38.2	45.0	19.0		scaffold_7:15646557:15646697:+
miR167a.2-3p	AGATCATCTGGCAGTTTCACC	21	21.8	1.2	10.9	1.2	1.2	0.6	8.3	0.6		scaffold_5:29592209:29592344:-
miR167a.2-5p	TGAAGCTGCCAGCATGATCT	20	14.3	6.7	28.9	20.5	23.2	24.7	56.8	17.4		scaffold_2:30397419:30397755:-<br>scaffold_5:29592209:29592344:-
miR167b-3p	AGATCATGCGGCAGTTTCACC	21	202.0	340.8	274.7	613.8	600.5	910.6	399.5	691.3		scaffold_9:4164049:4164180:+
miR167b-5p	TGAAGCTGCCAGCATGATCTTA	22	396.5	280.3	891.7	707.8	867.9	895.8	2026.9	836.8		scaffold_9:4164049:4164180:+
miR167c	TGAAGCTGCCAGCATGATCTA	21	20.1	5.7	45.4	18.2	56.8	30.8	83.0	20.2		scaffold_3:44075370:44075494:-
miR168	TCGCTTGGTGCAGGTCGGGAA	21	86.6	78.7	133.8	226.5	116.7	266.3	244.0	200.0		scaffold_5:29173067:29173242:-
miR169a.1	TAGCCAAGGATGACTTGCCT	20	3.4	0.9	2.7	5.0	5.0	3.2	1.0	1.3		scaffold_2:9780494:9780604:-<br>scaffold_2:12410778:12410936:-<br>scaffold_2:12410877:12411088:-<br>scaffold_2:12454738:12454859:-<br>scaffold_2:12475082:12475195:-<br>scaffold_2:12475375:12475506:-<br>scaffold_2:12521664:12521818:-<br>scaffold_4:13667210:13667327:+<br>scaffold_4:18890259:18890389:+<br>scaffold_4:24490875:24491077:+<br>scaffold_7:20296204:20296367:-<br>scaffold_8:18698592:18698722:+
miR169a.2	GCTAGCCAAGGATGACTTGCCT	22	3.4	1.0	2.8	2.4	1.8	0.8	0.9	0.3		scaffold_2:12454738:12454859:-
miR171a-5p	TGATTGAGCCGTGCCAATATC	21	13.6	5.2	11.5	14.9	22.9	11.8	14.7	11.7		scaffold_2:13369245:13369364:-<br>scaffold_7:477836:477993:-<br>scaffold_2:35379160:35379259:-<br>scaffold_4:12466830:12466929:+<br>scaffold_5:42334830:42334969:-

miR171c	TATTGGCCCGGTTCATTCAGA	21	3.6	9.1	5.5	33.6	10.4	56.8	10.9	28.7	scaffold_5:42334830:42334969:-
miR171d	TGAATGAACCGGGCCAATATC	21	0.4	0.2	0.2	0.5	1.2	0.7	0.6	0.4	scaffold_5:42334851:42334950:+
miR171e-3p	TTGAGCCGCGCCAATATCACT	21	0.1	0.2	0.9	0.0	0.4	0.8	0.2	0.6	scaffold_1:25748180:25748325:+
miR171g	CGAGCCGAATCAATATCACTC	21	0.7	1.2	0.5	4.1	1.1	1.7	0.9	1.3	scaffold_3:12772944:12773101:-
miR172a.1	GCAGCGTCCTCAAGATTCACA	21	26.7	27.4	19.2	25.8	31.9	22.9	19.0	8.3	scaffold_7:1319440:1319604:+
miR172b.1	AGAATCTTGATGATGCTGCA	20	1.5	0.4	0.6	1.6	0.3	0.5	0.2	0.2	scaffold_4:6541979:6542169:-
miR172c-3p	AGAATCTTGATGATGCTGCAT	21	1.2	0.1	0.8	0.7	1.1	0.5	0.6	0.5	scaffold_8:21551626:21551771:+
miR172c-5p	GTAGCATCATCAAGATTCAC	20	0.7	0.1	0.8	0.9	0.5	0.7	0.8	0.3	scaffold_8:21551626:21551771:+
miR172a.2	GCAGCGTCCTCAAGATTCACAT	22	0.3	0.2	0.2	0.1	0.3	0.2	0.0	0.1	scaffold_7:1319440:1319604:+
miR2275b-3p	TTTAGTTTCCTCCAATATCTTA	22	0.4	0.1	0.4	0.3	1.8	1.1	0.8	0.8	scaffold_6:24755603:24755735:+
miR2275b-5p	AGAATTGGATGGAACTAAACA	21	0.4	0.3	0.6	0.7	6.3	1.2	0.5	0.7	scaffold_6:24755603:24755735:+
miR319a.1	CTTGGACTGAAGGGAGCTCCT	21	10.8	4.4	7.4	9.8	30.5	20.8	22.5	11.3	scaffold_4:2444844:2445038:+<br>scaffold_5:14995166:14995360:+
miR319b	TTTGGACTGAAGGGAGCTCCT	21	834.1	265.9	1197.8	682.1	1521.7	853.8	2125.6	606.7	scaffold_1:4702941:4703034:-
miR319c.1	TTGGACTGAAGGGAGCTCCC	20	8.6	3.1	3.8	5.2	17.4	11.0	8.8	5.4	scaffold_3:1238941:1239168:+<br>scaffold_2:28033497:28033687:-
miR319c.2	CTTGGACTGAAGGGAGCTCCC	21	4.2	2.0	2.2	1.3	9.3	6.9	9.0	4.0	scaffold_3:1238941:1239168:+
miR319c.3	TTGGACTGAAGGGAGCTCCCA	21	7.8	1.3	2.9	1.9	13.0	8.0	6.2	3.2	scaffold_3:1238941:1239168:+
miR390	AAGCTCAGGAGGGATAGCGCC	21	102.8	91.3	99.3	211.8	236.7	342.3	197.7	234.2	scaffold_6:16983857:16984015:+<br>scaffold_8:24878571:24878733:+
miR393a.1	TCCAAAGGGATCGCATTGATCC	22	2.7	2.9	1.5	5.2	2.5	4.2	1.2	3.9	scaffold_2:8325011:8325111:+<br>scaffold_4:274425:274567:-
miR393a.2	TCCAAAGGGATCGCATTGATC	21	0.8	2.0	1.1	2.6	1.3	1.2	1.4	0.7	scaffold_2:8325011:8325111:+
miR393b-5p	TTCCAAAGGGATCGCATTGATC	22	7.8	5.8	4.9	11.6	7.7	7.3	8.4	7.7	scaffold_9:29029119:29029292:-
miR394a	TTGGCATTCTGTCCACCTCC	20	15.1	12.9	18.4	29.2	27.4	53.8	55.1	45.2	scaffold_5:42405317:42405488:+
miR395	CTGAAGTGTTTGGGGGAACTC	21	0.1	0.0	0.2	0.2	0.5	0.2	0.6	0.1	scaffold_3:38273180:38273272:+<br>scaffold_4:23647665:23647767:+
miR3951-3p	TTTCTCTTATCGTTATCTGTG	21	73.2	59.0	128.2	75.7	92.9	80.8	280.5	79.3	scaffold_1:20961000:20961168:+
miR3951-3p.2	TTTCTCTTATCGTTATCTGT	20	57.1	32.8	61.6	62.9	39.8	33.5	71.4	33.3	scaffold_1:20961000:20961168:+
miR3951-5p	TAGATAAAGATGAGAGAAAAA	21	53.4	71.0	68.8	129.2	52.9	133.4	71.2	138.5	scaffold_1:20961000:20961168:+
miR3952	TGAAGGGCCTTTCTAGAGCAC	21	979.4	358.6	1525.1	633.9	1261.5	630.2	2226.4	713.4	scaffold_8:17131560:17131766:+<br>scaffold_8:17214720:17214926:+
miR3954a	TTGGACAGAGAAATCACGGTCA	22	144.2	83.4	141.3	192.7	229.6	289.6	338.3	232.0	scaffold_9:5038089:5038264:+
miR3954b	TTGGACAGAGAAATCACGGTC	21	41.2	37.5	48.1	53.2	47.8	60.9	108.2	59.8	scaffold_9:5038089:5038264:+
miR396a	TTCCACAGCTTTCTTGAACTT	21	44.3	24.6	69.2	77.3	65.3	121.3	50.1	46.2	scaffold_1:25779169:25779340:+<br>scaffold_7:11659058:11659273:+
miR396b	TTCCACGGCTTTCTTGAACGT	21	1.5	1.8	1.5	8.9	0.6	0.2	0.4	0.9	scaffold_7:379001:379158:-

miR396c	TTCCACAGCTTTCTTGAACTG	21	11.2	14.6	20.1	20.4	7.4	21.8	14.5	17.7	scaffold_1:1677636:1677784:+<br>scaffold_1:25784771:25784918:-
miR396d	TTCCACGGCTTTCTTGAACTT	21	10.2	3.5	22.9	8.8	43.6	19.3	57.1	13.4	scaffold_4:18510610:18510780:-
miR397	TCATTGAGTGCAGCGTTGATG	21	0.8	1.4	1.0	2.6	3.7	4.8	3.5	3.3	scaffold_2:32706486:32706639:+
miR399a	CGCCAAAGGAGAATTGCCCTG	21	0.9	0.6	5.3	2.8	3.8	1.1	5.2	1.3	scaffold_2:34395812:34395944:+
miR399b	TGCCAAAGGAGAATTGCCCTG	21	0.2	0.0	0.6	0.5	0.7	0.1	0.5	0.1	scaffold_2:34395813:34395945:-
miR399c	TGCCAAAGGAGATTTGCCCGG	21	0.1	0.4	0.2	0.4	1.2	0.5	0.8	0.8	scaffold_2:34388563:34388655:-<br>scaffold_2:34401198:34401327:-
miR399d	TGCCAAAGGAGAGTTGCCCTA	21	2.3	1.2	4.2	10.6	3.8	2.1	7.1	2.2	scaffold_3:6735378:6735533:-
miR403	TTAGATTCACGCACAAACTCG	21	270.0	239.6	358.1	284.7	300.1	286.5	324.6	279.5	scaffold_3:46661847:46661976:-<br>scaffold_9:741537:741641:+
miR472	TTTTTCCCACACCTCCCATCCC	22	232.0	159.9	322.9	320.0	332.9	298.2	563.4	329.1	scaffold_2:28603077:28603170:-
miR473	ACTCTCCCTCAAGGGCTTCGC	21	9.7	5.0	10.1	19.2	18.3	10.1	15.2	10.2	scaffold_4:19101787:19101928:-
miR477a	ACTCTCCCTCAAGGGCTTCTGA	22	3.1	1.8	3.1	5.5	6.2	3.8	4.5	3.1	scaffold_3:1033183:1033381:-<br>scaffold_3:1033107:1033244:-
miR477b	ACTCTCCCTCAAGGGCTTCTGG	22	0.8	1.5	0.9	2.5	1.3	1.7	2.0	2.0	scaffold_3:1033319:1033467:-
miR477c	ACTCTCCCTCAAGGGCTTCTC	21	3.5	2.7	5.4	9.3	8.5	5.5	5.9	3.6	scaffold_4:19101972:19102126:-
miR479	TGTGATATTGGTTCGGCTCATC	22	0.9	0.3	2.1	2.6	1.4	1.0	1.4	1.4	scaffold_3:12772944:12773101:-
miR482a	TTGCCAACTCCTCCCATGCCGA	22	996.3	983.8	815.4	1262.4	2295.8	3007.4	2331.7	2574.6	scaffold_2:28637486:28637648:-
miR482b	TCTTGCCCACCCCTCCCATTCC	22	126.9	76.3	181.2	121.9	250.8	167.0	231.0	153.9	scaffold_2:28632294:28632439:-
miR482d	TCTTCCCTATGCCTCCCATTCC	22	375.6	167.3	615.6	448.8	536.0	491.5	859.8	463.1	scaffold_2:28622226:28622391:-
miR482e	TCTTACCTATGCCACCCATTCC	22	909.8	415.2	999.3	1021.0	1699.2	1230.2	2049.9	1077.4	scaffold_2:28618977:28619107:-
miR482f	TCCCTACTCCACCCATGCCATA	22	388.1	224.3	292.2	489.2	517.3	1005.2	465.7	605.2	scaffold_8:16726738:16726872:+
miR482g	TTCCCTAGTCCCCCTATTCCTA	22	123.3	122.0	98.5	168.3	312.2	408.0	269.6	389.8	scaffold_2:28637292:28637429:-
miR5179	TCTTGCTCAAGACCGCGCAAT	21	1.3	0.7	1.2	1.2	3.0	1.1	2.4	0.7	scaffold_3:30002351:30002607:+
miR530	TGCATTTGCACCTGCACCTTG	21	1.1	1.0	3.3	1.2	0.5	0.9	1.3	0.4	scaffold_8:21439576:21439699:+
miR535-3p	GTGCTCTCTACCATTGTCATA	21	0.6	0.4	1.8	2.6	2.7	6.2	5.9	5.6	scaffold_2:25552274:25552414:+
miR535-5p	TGACAATGAGAGAGAGCACAC	21	0.4	0.5	1.0	1.1	3.0	3.8	4.1	3.0	scaffold_2:25552274:25552414:+
miR827-3p	TTAGATGACCATCAACAAACA	21	0.4	0.4	0.9	0.4	24.9	9.4	52.4	9.0	scaffold_3:25587966:25588105:+
miR827-5p	CTTGTTGATTGTCATCTAATC	21	1.6	3.0	1.1	2.5	38.5	25.1	63.4	19.5	scaffold_3:25587966:25588105:+
miR828	TCTTGCTCAAATGAGTATTCCA	22	0.2	0.0	0.0	0.0	0.0	0.2	0.0	0.2	scaffold_3:12409080:12409226:-<br>scaffold_3:47964821:47964969:-
miR8747	TCACAGTTGTATTTAGTCGTT	21	0.3	0.2	0.6	0.3	0.7	0.5	0.5	0.4	scaffold_3:46690971:46691066:-
miR9560	ACAGGAGGTGGAACAAATATGAAA	24	7.1	2.5	19.4	4.3	19.2	25.4	46.8	21.9	scaffold_5:33719340:33719460:-
miRN01	TAGAACCTTTTAATTAATAAA	21	1.4	4.6	1.3	7.2	1.8	9.0	2.0	7.4	scaffold_3:51011258:51011413:-
miRN02	TATTATTGCAGTCGCCACATTC	22	0.4	0.3	0.9	0.3	5.1	4.0	4.3	4.2	scaffold_1:27119220:27119333:-
miRN03	GCTCTAAAGTGTTTGGTAAACACT	24	1.8	2.1	2.4	1.8	5.1	2.2	1.4	2.4	scaffold_7:11314734:11314940:+

miRN04	CTGATGAGAGAGCGAATGATA	21	0.4	0.0	0.2	0.5	7.5	5.5	8.2	2.6	scaffold_1:27997589:27997749:-
miRN05	TGTTTTGGGTGAAACGGGTGTT	22	4.2	2.6	7.6	5.6	5.9	5.1	10.8	6.1	scaffold_6:18972220:18972370:-
miRN06	AAATGCGGATTTGTTGTATACACC	24	161.8	183.1	160.4	118.6	148.7	98.1	147.7	119.8	scaffold_3:1838587:1838659:+
miRN07	ACGAGAGTTTGTGGCTGTATCATT	24	4.0	3.8	8.4	5.1	12.4	15.9	29.2	20.3	scaffold_3:7213370:7213585:+
miRN08	CCGTTTCATCTTGTCCTCCAG	21	170.0	68.3	157.1	137.4	400.0	271.9	405.3	187.8	scaffold_3:33172778:33172900:+<br>scaffold_3:33183546:33183668:+
miRN09	AGGGGCAATAAAGTGAATTCAAT	23	3.3	3.3	8.4	7.2	7.7	9.3	11.1	12.4	scaffold_3:9335031:9335159:-
miRN10	ACCGTAGGTGAACTCTAACATAGC	24	112.4	116.2	167.1	114.1	124.3	137.8	190.3	144.7	scaffold_3:50067748:50067961:-
miRN11	AGTTTCAGGATTGGTTTGGGATTC	24	6.7	6.6	4.6	7.6	11.6	7.7	5.9	8.5	scaffold_3:48438121:48438268:-
miRN12	TGAGAGACTGTACCTTACATGCA	23	2.2	3.4	1.6	3.4	3.0	4.2	2.7	2.9	scaffold_5:39064869:39064974:-
miRN13	AACATAATTGGGGAATAGATTCTC	24	15.8	12.4	8.7	12.4	17.6	13.7	10.6	17.4	scaffold_6:5283166:5283343:+
**miRN14**	ATTTTAAAACGTGCGGATGTCTGC	24	47.9	69.9	76.4	39.7	67.4	35.5	84.2	44.0	scaffold_7:19096789:19096982:+
**miRN15**	ACGTTATGGTGAGCGACACTGTCA	24	7.6	4.6	9.9	8.8	24.3	14.4	16.1	11.9	scaffold_5:6448011:6448176:-
miRN16	CACGCGGCCATCTCTCATTGA	21	0.2	1.3	0.2	4.4	0.7	9.5	0.1	5.6	scaffold_3:31161582:31161673:+
miRN17	TTTGATCTTGTCTTCCAGAGC	21	1.7	0.7	1.2	0.9	3.1	2.6	2.3	2.1	scaffold_3:33172783:33172884:-
miRN18	AGTTTCTGATAATATCTTTGCACC	24	18.2	10.4	13.4	22.0	0.5	0.5	0.1	0.6	scaffold_3:39754249:39754439:-
miRN19	AACAGCACCTAAAAGTTTCAACTG	24	1.3	2.3	1.1	3.2	1.4	3.3	1.4	3.1	scaffold_2:11898956:11899200:+
miRN20	TCAAGTAAGAGCGTTTTCTGTAAA	24	4.4	2.9	6.1	4.1	4.8	3.1	6.3	3.3	scaffold_5:2207028:2207200:+
miRN21	ACAGAATAGTAGTGAATTGATAAC	24	0.4	1.5	0.2	0.2	12.4	60.6	11.6	56.6	scaffold_5:7240594:7240927:-
miRN22	ATTTATACATGGAATTTAAATCTG	24	17.7	17.2	11.6	16.6	20.1	26.2	19.7	28.7	scaffold_5:39425102:39425462:-
**miRN23-3p**	TCGCAGGAGCTTTCTACGGTT	21	40.3	21.8	36.5	56.4	35.8	26.3	42.7	26.4	scaffold_8:17444229:17444445:+
**miRN23-5p**	CTGTAGAAGGCTCCTGTGACC	21	39.8	25.1	70.7	40.5	19.1	25.6	63.2	19.5	scaffold_8:17444229:17444445:+
miRN24	TTGAGAAGTGTAGTATTATTT	21	3.1	2.0	3.2	2.6	3.4	3.1	8.8	2.5	scaffold_8:2028426:2028638:-
**miRN25-5p**	CGTCCCCCTCACGGCTACAGTACC	24	49.0	17.8	84.1	33.8	120.8	71.9	181.4	77.3	scaffold_3:50071555:50071676:-
miRN25-3p	GTACTGTAGCTGTGAGGGGGACGT	24	88.4	31.5	101.1	60.4	28.3	32.1	15.4	38.3	scaffold_3:50071555:50071676:-
**miRN26**	TAAAATATTGAGTGAGAGAGGAAA	24	0.5	2.4	1.3	5.5	2.6	3.8	3.2	4.4	scaffold_9:22153707:22153831:+
miRN27	TTTCCGAGGGTGAAGGGACTT	21	2.9	2.0	3.2	4.1	10.2	6.7	6.0	3.5	scaffold_8:17214749:17214897:-
miRN28	TTGAGATTGAAAGTAGTGATT	21	24.3	22.7	41.4	45.6	64.5	64.2	85.2	33.8	scaffold_8:17461867:17462089:-
miRN29	CTTTGAACTCCTCGAAGCCT	20	26.3	14.2	31.3	18.1	29.2	15.7	67.8	13.3	scaffold_9:5534434:5534606:+
miRN30	TGGCTTTGCTCCACGAATCAT	21	32.6	17.2	24.4	40.8	54.6	46.4	35.9	38.6	scaffold_4:5046605:5046847:+
miRN31-5p	GGAGTGAACCTGAGAACAGAGG	22	0.2	0.8	0.6	1.1	2.1	22.9	1.6	3.5	scaffold_4:24311302:24311476:-
miRN31-3p	TTGTGTTCTCAGGTCACCCCT	21	0.1	0.1	0.0	0.2	7.2	12.3	5.8	6.7	scaffold_4:24311302:24311476:-
**miRN32**	ATTGGTCGGGTGCACCACCTCAGC	24	133.9	51.9	149.0	89.6	152.7	93.8	204.7	98.3	scaffold_8:8211928:8212175:+
**miRN33**	TCCTTAGTTGAGTTGGTTGGCATT	24	11.3	6.0	20.9	19.2	25.5	14.4	19.3	10.0	scaffold_6:16791570:16791806:+
miRN34	GATAATAAGAATTCTAATGGTACC	24	52.6	93.4	83.7	91.9	122.9	172.0	274.9	265.1	scaffold_2:10579110:10579344:+
miRN35	ACCGAATAAGAACCGAACTCAAAC	24	4.3	1.3	6.1	3.1	1.5	1.8	4.3	1.5	scaffold_2:22794667:22794747:-

**miRN36**	AAATCACTAACCGTCAAGTATAAG	24	13.7	12.0	10.0	27.6	21.8	36.5	13.2	32.1	scaffold_2:23904890:23905129:-
miRN37	CAAAGTCAAAAGTTGTATGTTAGC	24	3.2	3.1	3.2	3.2	23.1	22.1	36.8	25.8	scaffold_9:3248243:3248490:-
**miRN38**	TATACGTCGTTGTTCATGTATACT	24	33.4	28.4	26.7	37.6	80.9	73.7	54.0	65.6	scaffold_7:21115849:21116090:-
											scaffold_7:21117847:21118095:-
miRN39	TAGAGTTCAACTGTAATAATTGTT	24	3.8	5.2	3.1	6.0	5.6	11.9	4.8	12.9	scaffold_3:40948238:40948486:+
miRN40	ACACGGTACTATAGTTGCACC	21	8.8	4.8	9.6	6.3	8.7	3.7	10.3	2.0	scaffold_5:15799873:15800040:+
miRN41	TTTAAACTTCCGGATTAGAATATT	24	7.9	6.4	6.9	9.4	5.7	3.9	4.0	4.3	scaffold_3:47100815:47101052:+
miRN42	CTTTCACTCTTTTTGTTGCATG	22	3.7	2.0	4.8	3.3	7.1	5.8	9.2	4.2	scaffold_1:21925372:21925482:-
miRN43-3p	TCCCATCACCTTGGCATGCTGAGG	24	6.7	7.8	9.8	7.9	32.8	7.3	6.5	7.1	scaffold_3:7852222:7852421:-
miRN43-3p	AGCATGTCGAGGTGGTGGGATAGT	24	5.0	5.6	5.7	3.9	5.3	4.6	9.6	4.7	scaffold_3:7852222:7852421:-
**miRN44**	AACGAGTCACTTTTCTGTTAACTG	24	54.5	42.6	55.4	57.2	26.1	27.7	19.8	22.2	scaffold_2:7165151:7165397:-
**miRN45**	AACTTACACCAACTCACATGAACT	24	7.4	4.4	8.5	13.3	17.9	24.6	13.6	20.8	scaffold_3:40268808:40269001:-
miRN46	TACTGACGTGATATGATATGATTG	24	7.8	7.9	16.4	10.4	8.8	9.1	14.3	7.1	scaffold_9:1851239:1851408:+
miRN47	GACTGTCCCATCTAAGTTTTTCTC	24	5.6	8.0	5.0	10.8	5.4	11.7	7.5	12.8	scaffold_3:7852453:7852585:-
miRN48	AAAATTAGTAGGCGATGTTGTTTT	24	5.0	4.5	9.8	8.0	7.4	9.5	10.3	8.5	scaffold_8:13935087:13935310:-
miRN49	ATGACAGTGTTGCTCACTATAACG	24	1.3	0.8	1.8	2.5	1.8	1.8	3.1	2.3	scaffold_8:22716957:22717204:+
miRN50	AGCACGAGAGAAAGACGAGAGAAT	24	0.6	15.7	2.1	31.3	0.7	29.2	0.3	27.1	scaffold_2:7150061:7150179:+
miRN51	CTTTGAACTCCTCGAAGCCT	20	26.3	14.2	31.3	18.1	29.2	15.7	67.8	13.3	scaffold_9:5534389:5534637:-
miRN52	AAACAGCCACTGGAAATCTGCAGG	24	5.0	3.3	3.9	5.8	6.2	5.7	5.0	7.1	scaffold_5:8918489:8918613:-
miRN53	ACGACTGATATACCCTGAAAGTGT	24	3.2	4.3	3.6	4.1	1.3	1.7	1.7	1.5	scaffold_5:11473366:11473696:+
miRN54	AAGTACTTCCTTACACACACGGTA	24	1.4	0.7	1.4	0.8	3.4	5.4	6.7	6.4	scaffold_5:20239112:20239216:-
**miRN55**	ATAAAAAACACTCTCAAACGCACC	24	492.4	516.7	498.1	685.2	401.4	653.4	464.2	799.9	scaffold_7:1684361:1684440:-
**miRN56**	ATCTATTTGGATGAAGATAAGGGC	24	34.3	12.7	37.2	13.4	29.6	16.2	27.2	14.5	scaffold_4:611089:611222:+

The common novel miRNAs between two pairs of PU/GF and CM/PKare highlighted in bold.br1/br2: two biological replicates respectively.

Table S6 Differentially expressed miRNAs identified between the poly- and mono-embryonic ovules within cultivar pair

Name	pre-NEI		NEI	
	log2 (GF /PU)	FDR	log2 (GF /PU)	FDR
miR169a-3p	-3.35	0.0006413	-2.53	0.0016485
miR171b	3.98	1.8947E-05	8.22	4.343E-09
miR393b-3p	-1.66	0.0046757	-1.67	0.003901
miR393b-5p	-1.83	0.00011699	-1.83	0.0001041
miR3951-3p	7.43	1.4231E-30	7.20	1.518E-29
miR398	3.30	0.03576973	5.30	6.82E-03
miR399a	3.31	0.13151328	4.65	2.026E-06
miR399b	1.97	0.7792129	3.03	0.03738
miR399c	3.46	0.1343084	6.28	0.0031683
miR399d	1.85	0.07167528	2.02	0.0112498
miR473	0.72	0.59569014	1.80	0.0306943
miR477c	1.53	0.19027942	2.31	0.0112498
miR479	0.48	0.80045835	1.81	0.0211387
miR530	-1.79	0.01865067	-0.77	0.445169
miR9560	-0.62	0.87207041	-5.90	1.101E-05
miRN02-3p	5.24	3.5153E-12	6.09	1.721E-15
miRN02-5p	3.49	1.7477E-07	3.72	2.847E-08
miRN07	6.83	6.5127E-09	12.31	2.136E-13
miRN09	-5.37	8.6063E-08	-4.60	9.04E-07
miRN10	6.96	1.7007E-25	8.74	1.66E-31
miRN12	5.94	2.2596E-09	7.30	1.371E-12
miRN14-5p	-1.44	0.01393853	-1.17	0.0473496
miRN16	-8.69	1.0246E-08	-5.82	4.343E-09
miRN17	2.52	0.00319421	2.88	0.0004529
miRN20	8.40	1.218E-07	7.11	3.904E-10
miRN22	-1.75	0.09138193	-2.28	0.0138224
miRN23-3p	-1.79	1.782E-06	-1.99	8.155E-08
miRN23-5p	-1.66	5.4374E-05	-1.91	2.206E-06
miRN24	-1.73	0.04898934	-1.32	0.12731
miRN25	8.25	4.4436E-21	10.53	3.646E-25
miRN26	-0.12	0.97693515	-1.63	0.0449432
miRN28	-1.93	0.01393853	-2.55	0.0002386
miRN31	1.54	0.04881734	1.44	0.051941
miRN35	7.90	2.9965E-06	6.78	5.578E-10
miRN36	-1.23	0.0517488	-1.50	0.0086867
miRN37	-10.69	1.2E-17	-12.90	9.746E-29
miRN39	2.40	0.00371476	2.60	0.0012151
miRN43	-0.99	0.20554609	-1.50	0.0235354

miRN44	8.46	8.6354E-21	9.50	9.922E-22
miRN50	-9.10	1.3817E-27	-8.36	1.112E-25
miRN51	6.32	8.1898E-07	8.68	5.578E-10
miRN54	0.54	0.87207041	2.48	0.0473496
miRN55	1.97	1.8959E-05	1.97	1.382E-05
miRN59	6.38	1.5527E-07	10.30	5.286E-12
	**log2 (PK /CM)**	**FDR**	**log2(PK//PU)**	**FDR**
miR1446a	-8.71	3.424E-06	-6.51	2.747E-06
miR166e	1.64	0.1616371	2.07	0.0486791
miR167a.2-3p	-4.52	0.0133853	-0.98	0.8757856
miR169a.2	-1.70	0.2754530	-2.53	0.0486791
miR396b	-3.13	0.0573414	-3.25	0.0306682
miR827-3p	4.40	0.0010797	4.96	5.257E-05
miR827-5p	2.70	0.0133853	4.07	5.257E-05
miRN02	2.74	0.0259187	2.50	0.0306682
miRN04	4.11	0.0010797	3.56	0.0043875
miRN06	-1.52	0.0156334	-0.41	0.8672939
miRN18	-5.76	3.053E-06	-5.90	2.378E-06
miRN21	4.20	0.0053505	7.04	7.165E-06
miRN25-3p	-1.90	0.0416111	-1.80	0.0608851
miRN31-5p	3.57	0.0400128	1.31	0.8141012
miRN37	1.81	0.1032122	2.92	0.0013843
miRN44	-1.84	0.0133853	-1.73	0.0197477
miRN53	-2.33	0.0239387	-1.61	0.1937139
miRN54	1.06	0.6216164	2.21	0.0486791

FDR: false discovery rate.

Table S8 Differentially expressed miRNAs and their corresponding targets extracted from RNA-seq

miRNA	logFC(GF/PU) pre-NEI	logFC(GF/PU) pre-NEI	Target	logFC(GF/PU) pre-NEI	logFC(GF/PU) NEI	Annotation
miR171b	3.98	8.22	orange1.1t00200	-0.76	-0.21	gras family transcription factor
miR171b	3.98	**8.22**	Cs5g08980	-0.30	**-1.02**	scarecrow-like protein 27-like
miR171b	3.98	8.22	orange1.1t00199	-0.88	-0.13	gras family transcription factor
miR171b	3.98	**8.22**	Cs1g05390	-0.10	**-1.99**	protein tonsoku-like isoform x2
miR171b	3.98	8.22	Cs3g06940	0.11	-0.53	protein ethylene insensitive 3-like isoform x1
miR171b	3.98	8.22	Cs9g18700	0.37	0.37	hypothetical protein CICLE_v10005522mg
miR393b-3p	-1.66	**-1.67**	Cs5g31220	-0.89	**-2.25**	chalcone-flavanone isomerase family protein isoform partial
miR393b-5p	-1.83	-1.83	Cs2g14270	-0.30	0.94	protein auxin signaling f-box 2-like
miR393b-5p	-1.83	**-1.83**	orange1.1t02367	-0.60	**-1.51**	transcription factor bhlh62-like
miR393b-5p	-1.83	-1.83	Cs5g32500	0.26	-0.44	Protein TRANSPORT INHIBITOR RESPONSE 1
miR3951-3p	7.43	**7.2**	Cs2g03130	-0.77	**-1.23**	calcium-dependent protein kinase 21 isoform 1
miR3951-3p	7.43	7.2	Cs4g04820	-0.13	-0.13	probable starch synthase chloroplastic amyloplastic-like
miR3951-3p	7.43	7.2	Cs7g26090	0.84	0.84	serine threonine-protein kinase 16
miR3951-3p	7.43	**7.2**	Cs6g09130	0.18	**1.75**	NA
miR3951-3p	**7.43**	**7.2**	Cs1g23360	**1.71**	**1.19**	hypothetical protein CICLE_v10024678mg
miR3951-3p	**7.43**	**7.2**	Cs6g15280	**-1.22**	**1.19**	Pleiotropic drug resistance
miR3951-3p	7.43	7.2	Cs4g06350	0.53	0.64	polyadenylate-binding protein-interacting protein 7
miR3951-3p	7.43	7.2	Cs3g11210	-0.52	-0.78	disease resistance protein at4g27190-like
miR3951-3p	7.43	7.2	Cs8g01020	-0.06	-0.01	histone deacetylase 1 isoform 1
miR3951-3p	7.43	7.2	Cs3g16150	0.09	0.24	PREDICTED: uncharacterized protein LOC102628608 isoform X1
miR3951-3p	7.43	7.2	Cs1g08210	-0.46	-0.19	RNA exonuclease 4
miR398	3.3	5.3	Cs7g02980	-0.34	0.17	serine threonine-protein kinase pbs1
miR398	3.3	5.3	Cs3g12000	-0.97	-0.50	copper zinc superoxide dismutase
miR398	3.3	5.3	orange1.1t02346	-0.65	-0.07	calcium-transporting atpase plasma membrane-type-like
miR399b	1.97	3.03	orange1.1t03562	0.03	0.93	PREDICTED: uncharacterized protein LOC102630426 isoform X1
miR399c	3.46	**6.28**	Cs3g22510	0.62	**1.00**	protein sensitive to proton rhizotoxicity 1

miR399d	1.85	**2.02**	orange1.1t01536	0.88	**1.04**	alkaline neutral invertase isoform 1
miR473	0.72	**1.8**	Cs5g06850	-0.68	**-2.34**	della protein gai1-like
miR473	0.72	**1.8**	Cs5g03136	-0.71	**-1.01**	aaa-type atpase family protein
miR473	0.72	1.8	Cs3g19030	0.64	0.93	mitogen-activated protein kinase kinase kinase 1-like
miR477c	**1.53**	**2.31**	Cs8g17030	**2.28**	**4.70**	like cupins superfamily
miR477c	**1.53**	**2.31**	Cs3g10900	**1.11**	**1.17**	basic helix-loop-helix DNA-binding superfamily protein
miR479	0.48	1.81	Cs5g24810	0.12	0.39	exportin 1a isoform 1
miR530	-1.79	-0.77	Cs3g18550	-0.41	-0.81	pollen-specific protein c13-like
miR530	-1.79	-0.77	Cs9g08500	-0.05	-0.81	zinc knuckle family isoform 1
miR9560	-0.62	-5.9	Cs9g18000	-0.06	0.57	glutamate synthase 1
miRN02-3p	5.24	6.09	Cs9g10280	-0.67	-0.73	Uncharacterized protein At4g14342
miRN02-3p	5.24	**6.09**	Cs6g18560	-0.59	**-1.77**	Putative uncharacterized protein Sb01g043740
miRN02-3p	5.24	6.09	Cs5g32140	-0.50	-0.23	Histidine-containing phosphotransfer protein 5
miRN02-3p	5.24	6.09	Cs4g10920	0.14	0.45	RNA recognition motif family protein
miRN02-5p	3.49	3.72	Cs1g14600	-0.02	0.32	Coatomer subunit beta'-2
miRN12	5.94	**7.3**	Cs7g06370	0.43	**1.03**	Chorismate mutase, chloroplast, putative, expressed
miRN14-5p	**-1.44**	**-1.17**	Cs9g02390	**1.29**	**1.69**	Seed maturation protein PM36, putative, expressed
miRN14-5p	-1.44	-1.17	orange1.1t01197	-0.85	-0.45	Putative 40S ribosomal protein RPS26 (Fragment)
miRN16	-8.69	-5.82	Cs6g03820	-0.72	-0.11	Putative uncharacterized protein Sb01g038900
miRN16	**-8.69**	-5.82	Cs2g03220	**3.42**	0.37	Multi antimicrobial extrusion family protein
miRN16	-8.69	**-5.82**	orange1.1t00453	0.14	**1.10**	Common plant regulatory factor 1
miRN16	-8.69	-5.82	Cs3g16840	-0.27	-0.23	CBL-interacting serine/threonine-protein kinase 8
miRN16	-8.69	-5.82	Cs7g18010	-0.20	0.13	Putative uncharacterized protein OSJNBb0072E24.5
miRN17	2.52	2.88	Cs9g16710	0.57	0.95	Putative zfwd1 protein with similarity to myosin heavy chain proteins
miRN23-3p	**-1.79**	**-1.99**	Cs8g13560	**-4.17**	**-4.80**	/
miRN23-3p	**-1.79**	**-1.99**	Cs8g13550	**-1.58**	**-1.33**	Putative uncharacterized protein
miRN23-3p	-1.79	-1.99	Cs7g08020	0.17	0.91	SEC23-interacting protein
miRN23-3p	-1.79	-1.99	Cs7g24910	0.25	0.57	Transcription factor jumonji and C5HC2 type zinc finger domain-containing protei
miRN23-5p	**-1.66**	**-1.91**	Cs8g13550	**-1.58**	**-1.33**	Putative uncharacterized protein
miRN23-5p	-1.66	**-1.91**	orange1.1t00318	0.44	**1.22**	Digalactosyldiacylglycerol synthase 2, chloroplastic
miRN23-5p	-1.66	**-1.91**	Cs9g06920	0.84	**1.25**	Putative uncharacterized protein OJ1134F05.17
miRN35	7.9	**6.78**	orange1.1t01353	0.42	**-2.80**	Putative orcinol O-methyltransferase (Fragment)

miRN35	7.9	6.78	Cs9g19230	-0.20	0.06	Glutaminyl-peptide cyclotransferase
miRN36	**-1.23**	**-1.5**	Cs1g12520	**-2.67**	**-1.68**	Tetratricopeptide repeat domain-containing protein
miRN36	-1.23	**-1.5**	Cs8g06060	-0.03	**-2.70**	Thioredoxin M3, chloroplastic
miRN36	-1.23	**-1.5**	Cs1g23620	0.46	**1.10**	RING finger protein 5; E3 ubiquitin-protein ligase RMA1H1;
miRN37	**-10.69**	**-12.9**	Cs5g03640	**-7.77**	**-9.04**	Putative uncharacterized protein
miRN43	-0.99	-1.5	Cs3g24960	1.04	0.91	CBS domain-containing protein CBSX3, mitochondrial
miRN43	-0.99	**-1.5**	Cs2g12140	1.86	**-5.49**	Probably inactive leucine-rich repeat receptor-like protein kinase At5g4838
miRN50	-9.1	**-8.36**	Cs4g06980	0.43	**-1.95**	Invertase/pectin methylesterase inhibitor family protein / DC 1.2-like prote
miRN50	-9.1	-8.36	Cs1g22710	-0.53	-0.98	Pentatricopeptide repeat-containing protein At3g09040, mitochondrial
miRN50	-9.1	-8.36	Cs5g30670	0.45	0.76	Beige/BEACH and WD40 domain-containing protein
miRN51	6.32	8.68	Cs8g17820	-0.96	-0.75	Putative uncharacterized protein Sb04g020240
miRN51	6.32	**8.68**	Cs7g09080	-0.45	**-2.52**	Putative uncharacterized protein Sb03g004510
miRN54	0.54	**2.48**	Cs1g14690	5.30	**5.02**	Spore coat protein A
miRN54	0.54	**2.48**	orange1.1t04786	-3.08	**-3.65**	Spore coat protein A
miRN54	0.54	**2.48**	Cs7g04730	2.31	**2.29**	TMV resistance protein N;
miRN54	0.54	**2.48**	Cs8g15030	1.00	**2.04**	TGACG-sequence-specific DNA-binding protein TGA-2.1
miRN59	6.38	**10.3**	orange1.1t01353	0.42	**-2.80**	Putative orcinol O-methyltransferase (Fragment);
miRN59	6.38	10.3	Cs9g19230	-0.20	0.06	Glutaminyl-peptide cyclotransferase
miRN59	6.38	10.3	Cs6g18890	0.40	0.42	Putative uncharacterized protein

miRNA	logFC(PK/CM) pre-NEI	logFC(PK/CM) NEI	Target	logFC(PK/CM) pre-NEI	logFC(PK/CM) NEI	Annotation
miR1446a	-8.71	-6.51	Ciclev10024770m.g	0.94	0.43	autoinhibited Ca(2+)-ATPase 9
miR1446a	-8.71	-6.51	Ciclev10014097m.g	-0.37	-0.64	ARM repeat superfamily protein
miR1446a	-8.71	-6.51	Ciclev10000050m.g	-0.93	-0.10	Insulinase (Peptidase family M16) family protein
miR166e	1.64	**2.07**	Ciclev10023211m.g	0.85	**-1.23**	/
miR166e	1.64	**2.07**	Ciclev10014266m.g	-0.45	**-2.34**	Homeobox-leucine zipper family protein
miR166e	1.64	2.07	Ciclev10027790m.g	0.68	-0.36	Homeobox-leucine zipper family protein n
miR166e	1.64	2.07	Ciclev10007435m.g	0.01	-0.66	homeobox gene 8
miR166e	1.64	2.07	Ciclev10014947m.g	0.58	-0.44	cytochrome P450, family 71, subfamily B, polypeptide 34
miR166e	1.64	2.07	Ciclev10007477m.g	0.00	0.27	CRS1 / YhbY (CRM) domain-containing protein
miR166e	1.64	2.07	Ciclev10031260m.g	-0.44	-0.11	Dihydropterin pyrophosphokinase / Dihydropteroate synthase
miR166e	1.64	2.07	Ciclev10025269m.g	0.79	-0.64	asparagine synthetase 3

miR166e	1.64	2.07	Ciclev10000330m.g	-0.22	-0.43	/
miR166e	1.64	**2.07**	Ciclev10000097m.g	-0.60	**-1.81**	ATP binding microtubule motor family protein
miR167a.2-3p	**-4.52**	-0.98	Ciclev10009236m.g	**-1.79**	-0.29	calcineurin B-like protein 10
miR167a.2-3p	-4.52	-0.98	Ciclev10004460m.g	-0.95	-0.10	long-chain acyl-CoA synthetase 7
miR167a.2-3p	-4.52	-0.98	Ciclev10013939m.g	0.31	-0.29	/
miR167a.2-3p	-4.52	-0.98	Ciclev10013938m.g	0.31	-0.29	/
miR169a.2	**-1.7**	**-2.53**	Ciclev10032767m.g	**3.27**	**5.22**	HMG-box (high mobility group) DNA-binding family protein
miR827-3p	**4.4**	4.96	Ciclev10019105m.g	**-1.42**	-0.44	Major Facilitator Superfamily with SPX (SYG1/Pho81/XPR1) domain-con
miR827-3p	4.4	**4.96**	Ciclev10016360m.g	0.69	**1.02**	Ribosomal L18p/L5e family protein
miR827-3p	4.4	4.96	Ciclev10019641m.g	-0.25	0.29	Major facilitator superfamily protein
miR827-3p	4.4	4.96	Ciclev10028859m.g	0.93	-0.60	methyl-CPG-binding domain 7
miR827-3p	4.4	**4.96**	Ciclev10004479m.g	-0.01	**-2.29**	Phototropic-responsive NPH3 family protein
miR827-3p	4.4	4.96	Ciclev10027242m.g	0.93	-0.94	Tesmin/TSO1-like CXC domain-containing protein
miR827-5p	2.7	4.07	Ciclev10000010m.g	0.53	-0.05	Armadillo/beta-catenin-like repeat
miR827-5p	2.7	**4.07**	Ciclev10027792m.g	0.34	**-1.27**	Protein of unknown function (DUF630 and DUF632)
miR827-5p	2.7	4.07	Ciclev10030589m.g	-0.96	-0.17	histidine kinase 3
miR827-5p	2.7	4.07	Ciclev10020940m.g	0.99	0.77	Emsy N Terminus (ENT)/ plant Tudor-like domains-containing protein
miR827-5p	2.7	4.07	Ciclev10004835m.g	0.34	0.01	Protein of unknown function (DUF1423)
miR827-5p	2.7	4.07	Ciclev10004294m.g	0.55	-0.31	Nucleoporin interacting component (Nup93/Nic96-like) family protein
miRN02	**2.74**	2.5	Ciclev10003639m.g	**-1.30**	0.11	recovery protein 3
miRN02	2.74	2.5	Ciclev10015197m.g	-0.75	-0.16	transferases;nucleotidyltransferases
miRN02	2.74	2.5	Ciclev10018802m.g	0.80	0.91	Leucine-rich repeat protein kinase family protein
miRN02	2.74	2.5	Ciclev10020407m.g	0.65	-0.07	esterase/lipase/thioesterase family protein
miRN04	4.11	3.56	Ciclev10021235m.g	-0.70	-0.04	ARM repeat superfamily protein
miRN04	4.11	3.56	Ciclev10019952m.g	0.16	0.17	Leucine-rich repeat (LRR) family protein
miRN04	4.11	3.56	Ciclev10000551m.g	0.57	0.66	DNAJ heat shock N-terminal domain-containing protein
miRN04	4.11	3.56	Ciclev10025309m.g	-0.35	-0.20	Major facilitator superfamily protein
miRN04	**4.11**	**3.56**	Ciclev10032089m.g	**-1.22**	**-1.26**	PLATZ transcription factor family protein
miRN04	**4.11**	**3.56**	Ciclev10014378m.g	**1.53**	**1.19**	ERD (early-responsive to dehydration stress) family protein
miRN04	**4.11**	**3.56**	Ciclev10014696m.g	**1.53**	**1.19**	ERD (early-responsive to dehydration stress) family protein
miRN18	-5.76	-5.9	Ciclev10024987m.g	-0.32	0.52	ARM repeat superfamily protein
miRN18	-5.76	**-5.9**	Ciclev10007367m.g	0.14	**1.37**	H(+)-ATPase 5

miRN23-5p	-5.76	-5.9	Ciclev10030076m.g	-0.86	-0.96	/
miRN23-5p	**-5.76**	-5.9	Ciclev10023521m.g	**-1.02**	0.10	digalactosyl diacylglycerol deficient 2
miRN23-5p	-5.76	-5.9	Ciclev10005503m.g	-0.67	0.09	/
miRN31-5p	3.57	1.31	Ciclev10025231m.g	-0.16	0.37	DEAD-box protein abstrakt, putative
miRN31-5p	3.57	1.31	Ciclev10021146m.g	0.70	-0.14	Transducin/WD40 repeat-like superfamily protein
miRN31-5p	3.57	1.31	Ciclev10021567m.g	0.19	-0.07	Peptidase C13 family
miRN31-5p	3.57	1.31	Ciclev10024315m.g	0.33	0.28	Peptidase C13 family
miRN31-5p	3.57	1.31	Ciclev10014653m.g	0.25	-0.30	O-fucosyltransferase family protein
miRN31-5p	**3.57**	1.31	Ciclev10004265m.g	**-1.12**	0.35	Leucine-rich repeat transmembrane protein kinase
miRN31-5p	3.57	1.31	Ciclev10016199m.g	0.50	0.25	5\'-AMP-activated protein kinase beta-2 subunit protein
miRN31-5p	3.57	1.31	Ciclev10004184m.g	0.01	0.24	BRCT domain-containing DNA repair protein
miRN53	**-2.33**	**-1.61**	Ciclev10002302m.g	**-1.46**	**-1.35**	Nodulin MtN3 family protein

Note: The negative correlation between miRNA and target is highlighted in red, while the positive correlation between miRNA and target is highlighted in bold.
log2FC: log2 fold change.
